# PAM-free hairpin target binding activates ***t******rans***-cleavage activity of Cas12a

**DOI:** 10.1093/nar/gkaf596

**Published:** 2025-06-25

**Authors:** Xiaolong Li, Zixuan Zhu, Jiani Wu, Changjiang Li, Zhujun Liu, Jinjin Wang, Pu Li, Zhen Zhang, Yongming Huang, Jiaxin Hong, Tongbo Wu

**Affiliations:** School of Pharmacy, Tongji Medical College, Huazhong University of Science and Technology, Wuhan, 430030, China; School of Pharmacy, Tongji Medical College, Huazhong University of Science and Technology, Wuhan, 430030, China; School of Pharmacy, Tongji Medical College, Huazhong University of Science and Technology, Wuhan, 430030, China; School of Pharmacy, Tongji Medical College, Huazhong University of Science and Technology, Wuhan, 430030, China; School of Pharmacy, Tongji Medical College, Huazhong University of Science and Technology, Wuhan, 430030, China; School of Pharmacy, Tongji Medical College, Huazhong University of Science and Technology, Wuhan, 430030, China; School of Pharmacy, Tongji Medical College, Huazhong University of Science and Technology, Wuhan, 430030, China; The First Affiliated Hospital, and College of Clinical Medicine of Henan University of Science and Technology, Luoyang, 471003, China; Department of Gastrointestinal Surgery, Union Hospital, Tongji Medical College, Huazhong University of Science and Technology, Wuhan, 430022, China; Cancer Center, Union Hospital, Tongji Medical College, Huazhong University of Science and Technology, Wuhan, 430022, China Hubei Province Key Laboratory of Precision Radiation Oncology, Wuhan, 430022, China; Institute of Radiation Oncology, Union Hospital, Tongji Medical College, Huazhong University of Science and Technology, Wuhan, 430022, China; Hubei Province Key Laboratory of Precision Radiation Oncology, Wuhan, 430022, China; School of Pharmacy, Tongji Medical College, Huazhong University of Science and Technology, Wuhan, 430030, China

## Abstract

CRISPR–Cas12a has been demonstrated to be activated for its *trans*-cleavage activity by single- and double-stranded DNA containing a protospacer adjacent motif (PAM), but other types of activators have remained undiscovered. In this work, we found that a hairpin-structured substrate can activate the *trans*-cleavage activity of Cas12a without a PAM, and the parameters of the hairpin loop obviously affect the activity. Cas12a exhibits sequence preference for proximal loops, preferring to recognize polyadenine hairpin loop activators. Molecular docking and dynamic calculations provide a theoretical basis for the activation of Cas12a by hairpin activators. Leveraging the efficient activation capability of the hairpin activator, we constructed an allosteric detection platform for non-nucleic acid targets, capable of sensitively and specifically detecting hypochlorous acid and calcium ions. This novel activator of Cas12a holds enormous potential for the development of multi-functional biological platforms.

## Graphical Abstract

**Graphical Abstract:**
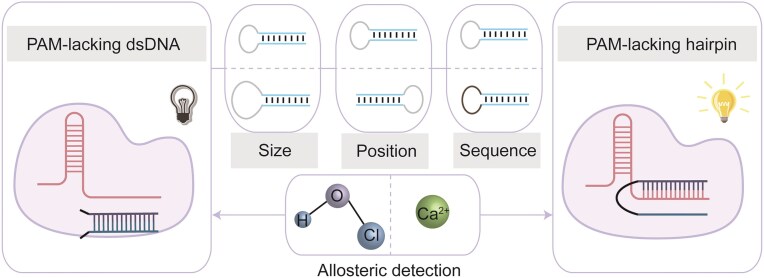

## Introduction

The CRISPR–Cas [clustered regularly interspaced short palindromic repeats (CRISPR)–CRISPR-associated protein] technology has significantly propelled the advancement of the gene editing field and is increasingly being utilized in molecular diagnostics [1–3]. The core of CRISPR–Cas12a technologies lies in the target-specific activation of nuclease activity, particularly the *trans*-cleavage activity. Specifically, upon recognition and cleavage of the target by the Cas12a/CRISPR RNA (crRNA) ribonucleoprotein (RNP), a protein conformational change enables the cleavage of arbitrary single-stranded DNA (ssDNA) near the exposed functional domain [4, 5].

Previous research indicates that double-stranded DNA (dsDNA) containing a protospacer adjacent motif (PAM, 5′-TTTV-3′) or ssDNA can activate Cas12a [6]. For the dsDNA activator, the activation relies heavily on the recognition of the PAM sequence by the slit between the wedge (WED), recognition (REC), and PAM-interacting (PI) domains of Cas12a, which induces the insertion of lysine into the dsDNA, triggering the unwinding of the dsDNA. This allows the target strand (TS) of dsDNA to form an RNA/DNA heteroduplex with crRNA, and an R-loop structure with the non-target strand (NTS), leading to nuclease activity through a process of conformational changes in the Cas12a protein [5]. Building on these facts, some classic Cas12a-based target detection strategies have been proposed. Doudna *et al.* developed the DNA endonuclease-targeted CRISPR trans reporter (DETECTR) system, which utilizes recombinase polymerase amplification (RPA) to amplify target nucleic acids, yielding PAM-containing dsDNA targets capable of activating Cas12a [7]. The activated Cas12a non-specifically cleaves the signal reporter to generate a fluorescent detection signal. Similarly, Wang *et al.* constructed the one-hour low-cost multipurpose highly efficient system (HOLMES) diagnostic strategy, incorporating the PAM sequence into the primer design [8]. During PCR amplification, the PAM sequence is introduced into the amplified product, thereby activating Cas12a and accomplishing the detection objective. Moreover, for the ssDNA activator, the target can directly bind with the crRNA to form an RNA/DNA heteroduplex, thus eliminating the need for a PAM sequence. Therefore, the detection strategies are often accompanied by the release or polymerization of the ssDNA activator. Lu *et al.* designed an allosteric probe that can release the originally locked ssDNA activator to activate the *trans*-cleavage activity of Cas12a with a specific molecular target [9]. Additionally, Yan *et al.* reported a one-pot isothermal method for the accurate detection of specific microRNAs. While Cas12a is activated by rolling circle amplification (RCA) products, the *cis-*cleavage activity of Cas12a enables exponential RCA amplification of the target sequence and the *trans*-cleavage activity for detection and signal amplification [10].

For non-canonical activators, studies have also reported that split dsDNA or ssDNA targets can still meet the activation requirements of Cas12a. The distal split activator can be dsDNA, ssDNA, or even RNA, but the proximal split activator can only be dsDNA containing a PAM or ssDNA [11–14]. Furthermore, it has been verified that small circular substrates cannot successfully activate Cas12a, which has spurred the construction of an autocatalytic amplification system, achieving a highly sensitive strategy without pre-amplification [15, 16]. However, the currently active activators of Cas12a ultimately remain at dsDNA containing a PAM or ssDNA. Regardless of whether it is a classical or non-canonical activator, as long as the front end of the seed region is a duplex structure, it is difficult to avoid PAM-assisted unwinding and binding. Although some studies claimed that the activation could be achieved without a PAM, they almost all utilize strand displacement to obtain the required ssDNA activator, which heavily depends on the toehold-mediated thermodynamic drive [17–20]. Specifically, in addition to amplifying dsDNA activators containing a PAM through primer design, almost all of them release locked ssDNA activators from the strand displacement reaction, and even introduce artificial unwinding structures such as bubbles in the seed region. However, these are not intrinsically PAM-free activations by dsDNA, but rather achieve the conversion from inactive PAM-lacking dsDNA molecules to active ssDNA activators. Direct activation independent of a PAM or toehold reaction proposed by our work can provide a new activation paradigm. It remains unclear whether other conformational substrates can directly endow Cas12a with *trans*-cleavage activity, or exhibit a specific preference in substrate recognition. Given that the activation characteristics of Cas12a by substrates without a PAM can broaden its application range, further characterization of the activation properties of PAM-free substrates on Cas12a and the development of PAM-free strategies based on Cas12a are of great significance in the field of biosensing and other related areas.

Herein, we report a hitherto undiscovered Cas12a active substrate, a hairpin (Hp)-structured DNA activator, which is composed of a loop (arbitrary sequence) and a stem (protospacer dsDNA). The loop at the terminus of the dsDNA allows for the activation of Cas12a's *trans*-cleavage activity without the requirement for a PAM sequence. Based on a series of sequence and structural analyses, we demonstrate that the Hp-structured DNA substrate can serve as a new type of activator for Cas12a, functioning independently of PAM assistance and thus representing a novel functional tool. As a proof of concept, we have employed a Hp-to-dsDNA allosteric strategy, wherein the loop region is targeted to enable direct detection of hypochlorous acid (HOCl) and Ca^2+^. Notably, the distinct properties of the Hp-structured DNA activator permit rational design tailored to specific requirements. More importantly, this represents a truly PAM-free activation of dsDNA via a loop structure, which has the potential to break through the boundaries of CRISPR–Cas12a system technology and enhance its accessibility.

## Materials and methods

### Materials and instruments

All oligonucleotides (Supplementary Table S1) were synthesized and purified by Sangon Biotech (Shanghai, China) and diluted with 1× TE buffer (10 mM Tris-HCl, 1 mM EDTA, pH 7.8) from Sangon Biotech. The concentrations of DNA oligonucleotides were measured by a NanoDrop 2000 UV-vis Spectrophotometer (ThermoFisher Scientific, MA, USA). LbCas12a, 10× NEBuffer r2.1 (10 mM Tris–HCl, 10 mM MgCl_2_, 50 mM NaCl, 100 μg/ml recombinant albumin, pH 7.9), 10× NEBuffer r3.1 (50 mM Tris–HCl, 10 mM MgCl_2_, 100 mM NaCl, 100 μg/ml recombinant albumin, pH 7.9), and 10 × NEBuffer 4 [20 mM Tris-acetate, 10 mM magnesium acetate, 50 mM potassium acetate, and 1 mM dithiothreitol (DTT), pH 7.9] were all obtained from New England Biolabs (NEB, MA, USA). AsCas12a was obtained from Genscript Biotech Corporation (Nanjing, China), and FnCas12a was purchased from Beyotime Biotech (Shanghai, China). Calcium chloride (CaCl_2_), magnesium chloride hexahydrate (MgCl_2_·6H_2_O), zinc sulfate (ZnSO_4_), and strontium chloride (SrCl_2_) were purchased from BBI Life Science. Lead chloride (PbCl_2_) and manganese chloride (MnCl_2_) were purchased from Sangon Biotech. Deionized water (DNase/RNase free) was obtained from Tiangen Biotech (Beijing, China) and used in all experiments. The fluorescence curves of all reaction solutions were recorded by a Rotor-Gene Q real-time PCR instrument (QIAGEN, Hilden, Germany) in the green channel (470 nm for excitation wavelength, 525 nm for emission wavelength).

### *Trans*-cleavage activity assay of activators with different structures

Hp activators and dsDNA activators (TS:NTS = 1:1.2) were both pre-annealed in 1× TM buffer (1 mM Tris–HCl, 0.05 mM MgCl_2_, pH 8.0) to obtain a final concentration of 250 nM. The annealing procedure involved heating at 95°C for 5 min, followed by cooling at 65°C for 5 min, and then at 37°C for 5 min. Subsequently, 2 μl of the 250 nM activator was added to the reaction mixture, which included 6 μl of Cas12a RNP pre-incubated at 37°C for 15 min (comprising 2 μl of 10× NEBuffer r2.1, 2 μl of 1 μM crRNA, and 2 μl of 500 nM Cas12a), 2 μl of 1 μM reporter, and 10 μl of deionized water. The real-time fluorescence at 37°C was immediately measured using the Rotor-Gene Q real-time PCR instrument, with the green channel and the 5 gain value.

### Hypochloric acid detection assays

We selected a Hp activator with a distal loop as the substrate for HOCl. The same annealing procedure was applied to the Hp substrate with a phosphorothioate-modified loop to avoid secondary structures. Sodium hypochlorite solution was acidified with sulfuric acid to form an HOCl solution. A 2 μl aliquot of the 250 nM Hp substrate and 2 μl of HOCl at various concentrations were mixed in 8 μl of deionized water and incubated at 37°C for 1 h. Subsequently, 6 μl of pre-incubated Cas12a RNP (2 μl of 10× NEBuffer r2.1, 2 μl of 1 μM crRNA, and 2 μl of 500 nM Cas12a) and 2 μl of 1 μM reporter were added. Real-time fluorescence at 37°C was immediately measured using the Rotor-Gene Q real-time PCR instrument, with the green channel selected and the gain value set to 5.

### Calcium ion detection assays

We selected a Hp activator with a proximal loop as the substrate for the Ca^2+^-dependent DNAzyme. The Hp substrate containing adenine ribonucleotides (rAs) as cleavage sites was first mixed with EtNa DNAzyme at a 1:1 ratio and annealed in 1× buffer (2.5 mM Tris–HCl, 20 mM NaCl) at 95°C for 5 min, 65°C for 5 min, and then at 37°C for 5 min, to obtain a 250 nM Hp–DNAzyme complex. Similarly, 2 μl of the 250 nM Hp–DNAzyme complex and 2 μl of CaCl_2_ solution at various concentrations were mixed in 8 μl of deionized water and incubated at 37°C for 1 h. Subsequently, 6 μl of pre-incubated Cas12a RNP (2 μl of 10× NEBuffer r2.1, 2 μl of 1 μM crRNA, and 2 μl of 500 nM Cas12a) and 2 μl of 1 μM reporter were added. Real-time fluorescence at 37°C was immediately measured using the Rotor-Gene Q real-time PCR instrument, with the green channel selected and the gain value set to 5. In the selectivity experiments, different ion solutions were used to replace CaCl_2_ at the same volume for validation. In the spike recovery experiments, actual water samples were used to replace deionized water for the same procedure. In the true-on mode, the single-stranded reporter was replaced with an invader strand for equivalent operation. Subsequently, the double-stranded reporter was introduced to generate the signal output.

## Results

### PAM-free hairpin activates the *trans*-cleavage activity of Cas12a

**Figure 1.**
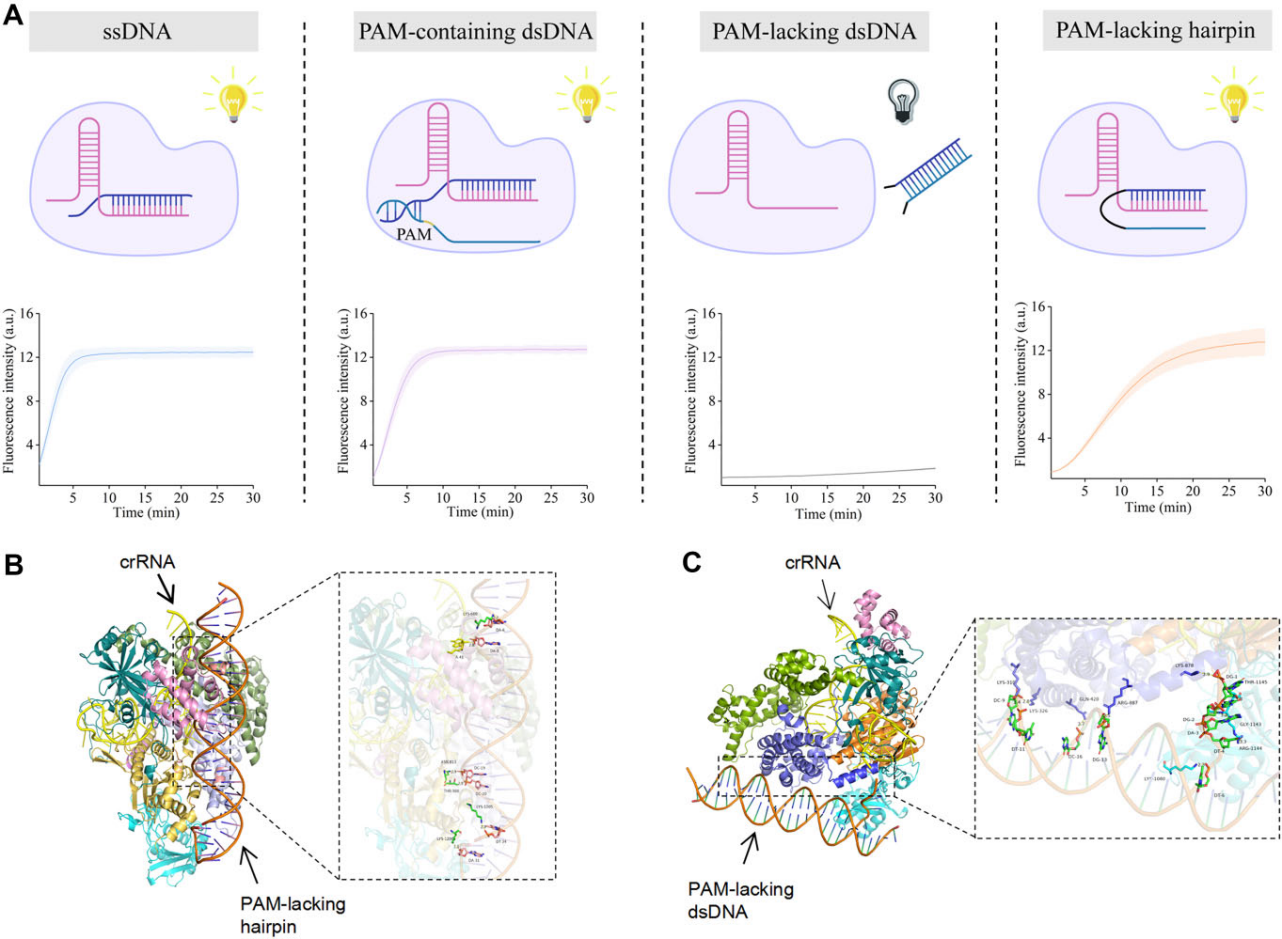
PAM-independent hairpin substrate enables Cas12a to exhibit *trans*-nuclease activity. (**A**) Activation of Cas12a *trans*-cleavage activity by different substrates: ssDNA, PAM-containing dsDNA, PAM-lacking dsDNA, and PAM-lacking hairpin. (**B**) Molecular docking between PAM-lacking hairpin substrate and Cas12a/crRNA RNP. (**C**) Molecular docking between PAM-lacking dsDNA substrate and Cas12a/crRNA RNP. Error bar, SD, *n* = 3.

We initially compared the efficiency of traditional ssDNA, PAM-containing dsDNA, PAM-lacking dsDNA, and PAM-lacking Hp in activating Cas12a. All four types of substrates were fully complementary to the 24 nt spacer of the crRNA, with only structural differences, without sequential differences in the protospacer region. The *trans*-cleavage activity of CRISPR–Cas12a was characterized using a 15 nt polythymine (poly-T) ssDNA reporter, modified with a fluorophore (FAM) and a quencher (BHQ-1) at both ends [21]. The experimental results indicated that the PAM-lacking Hp activator could effectively activate the *trans*-cleavage activity of Cas12a. Although the fluorescence increasing rate was slightly lower than that of the canonical ssDNA and PAM-containing dsDNA, it still achieved the same degree of fluorescent signal within 30 min. In contrast, the loop-disrupted PAM-lacking Hp (referred to as PAM-lacking dsDNA) could hardly activate the *trans*-cleavage activity of Cas12a (Fig. 1A; Supplementary Fig. S1). The HDOCK program was employed to perform molecular docking between DNA and protein–RNA complexes predicted by AlphaFold3. The selected complex structures were subjected to interaction analysis and visualized using PyMOL (Version 3.0.3) [22–24]. For the PAM-lacking Hp, partial hydrogen bonds were observed between the Hp and Cas12a or the Hp and the crRNA within the RNP (Fig. 1B). However, no interactions were observed between the PAM-lacking dsDNA and the crRNA (Fig. 1C). This lack of interaction affects the unwinding of the double-stranded protospacer region, which is a crucial factor for the activation of Cas12a.

**Figure 2.**
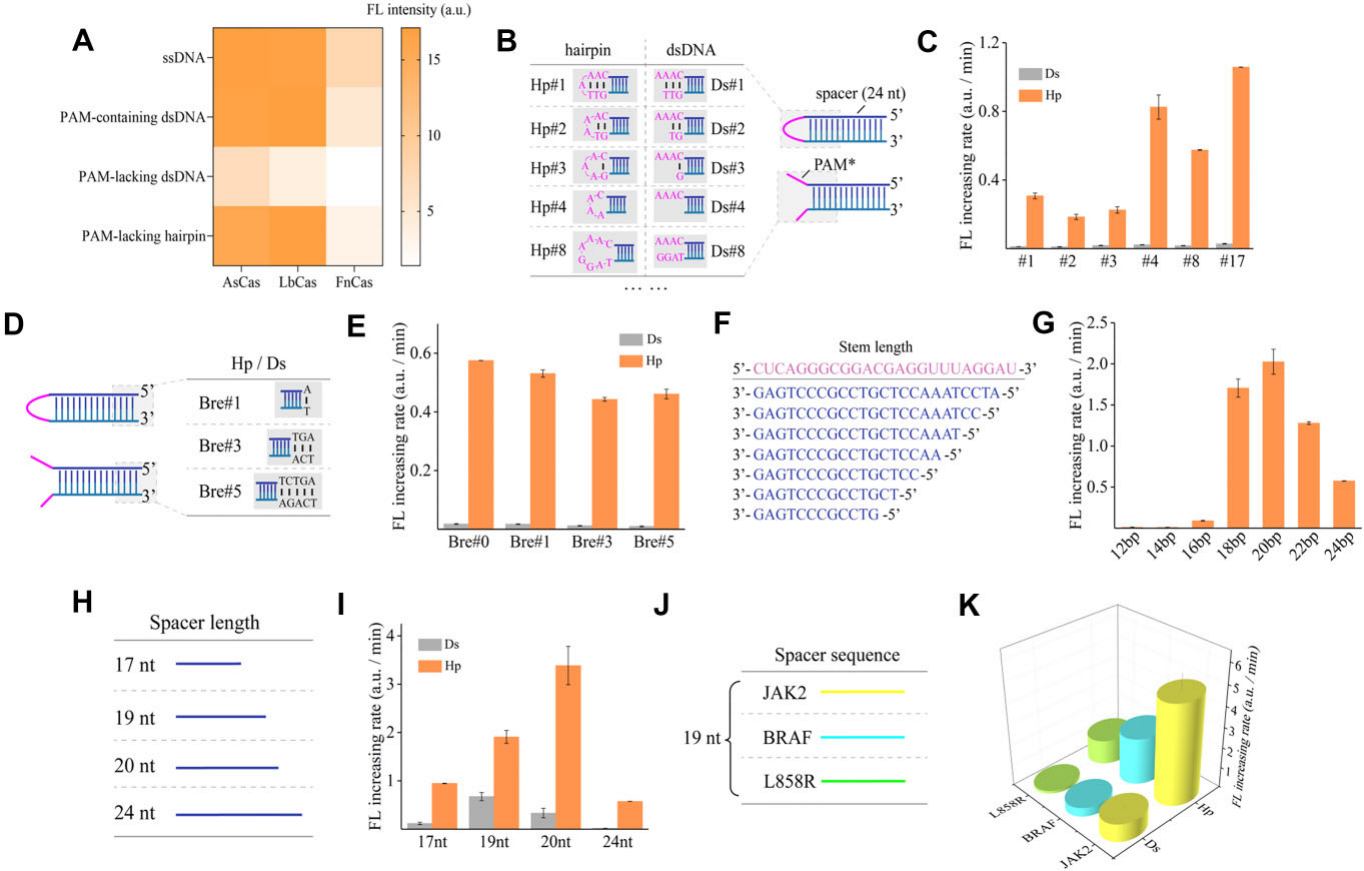
Analysis of *trans*-cleavage activity based on differences in Hp structure. (**A**) The activation differences of four types of substrates on Cas12a orthologs, compared based on the fluorescence intensity of the cleaved reporter at 30 min. (**B**) Schematic of PAM-free Hp and dsDNA substrates with various numbers of bases (PAM*) concealed within the protospacer adjacent region of the loop or ds/ss structure. (**C**) Measurement of the *trans*-cleavage activity of Cas12a for different numbers of bases in the protospacer adjacent region. (**D**) Schematic of #8 variants with different extension of the stem at the distal end. (**E**) Comparison of signals with different extension at the distal end of the stem. (**F**) Schematic showing the positioning of different stem lengths of Hp in the protospacer region, gradually shortening from the distal end of the PAM. (**G**) Results of the *trans*-cleavage activity of Cas12a activated by Hps with different stem lengths. (**H**) Schematic of variable spacer length of crRNA, adapting to perfectly matched substrates. (**I**) Results of the *trans*-cleavage activity of Cas12a activated by substrates corresponding to different spacer lengths. (**J**) Investigation of the variable spacer sequence of crRNA with a 19 nt spacer region, adapting to perfectly matched substrates. (**K**) Comparison of the *trans*-cleavage activity of Cas12a between the Hp and dsDNA substrate with the same spacer length but different spacer sequences. Represented as the fluorescence increasing rate. Error bar, SD, *n* = 3.

To illustrate the universality conferred to Cas12a's *trans*-cleavage activity by the Hp-structured activator, we conducted validations across three common Cas12a orthologs (AsCas12a, LbCas12a, and FnCas12a, derived from *Acidaminococcus* sp. BV3L6, *Lachnospiraceae bacterium* ND2006, and *Francisella novicida* U112, respectively) [25, 26]. We designed the sequence of PAM-lacking Hp substrate with a PAM-complementary sequence (PAM*, 5′-CAAA-3′) in the loop region which is commonly encountered in research [27], and other loop sequences were randomly designed. For the PAM-lacking dsDNA, a nick was designed at a fixed position in the loop of the PAM-lacking Hp to obtain the TS with PAM* and the NTS without PAM. Regardless of the Cas12a orthologs, the Hp-structured activator exhibited a slight disparity in activation efficiency compared with the canonical substrate but was significantly higher than that of the PAM-lacking dsDNA (Fig. 2A; Supplementary Fig. S2). Similarly, this phenomenon also applies in different buffers, where the activation efficiency of the Hp-structured activator is always higher than that of PAM-lacking dsDNA (Supplementary Fig. S3).

Considering that the stability of the Hp might be one of the primary factors, we conducted a detailed analysis of the PAM-lacking Hp. Taking LbCas12a as an example, we designed loops of 1, 2, 3, 4, 8, and 17 nt in size (#1, #2, #3, #4, #8, and #17), along with corresponding PAM-lacking dsDNA (Ds) for comparison (Fig. 2B). The activation efficiency of the Hp was consistently higher than that of the Ds, and a high activation efficiency was achieved when the loop size of Hp was > 4 nt (Fig. 2C). To weaken the DNA breathing dynamics (Bre), we extended the stem of the Hp by 1, 3, and 5 bp at the distal end (Bre#1, Bre#3, and Bre#5) with an 8 nt loop size (Fig. 2D). Only a slight decrease of the activation efficiency was observed, indicating that the main activation effect originates from the loop of Hp (Fig. 2E).

Previous studies have shown that the affinity between Cas12a RNP and activators significantly affects the activation efficiency of the activator, in which the GC content and length of the protospacer are direct manifestations of differences in affinity [11–14, 28, 29]. We designed Hps with different lengths of protospacer with a stem length of 12, 14, 16, 18, 20, 22, and 24 bp (Fig. 2F). As shown in Fig. 2G, a protospacer longer than 18 bp could efficiently activate Cas12a and reaches the maximum at 20 bp, and the results were similar to those of the canonical duplex activation substrates. The influence of the relative position between the crRNA spacer and substrate has been explained in previous studies, where a crRNA 3′ overhang leads to accumulation of conserved aromatic residues at the end of the R-loop, hindering the extension of the R-loop and thus affecting the activity of Cas12a [30]. Additionally, we designed crRNAs with 17, 19, 20, and 24 nt spacer lengths and the correspondingly matched stem sequences of Hp (Fig. 2H). As shown in Fig. 2I, the spacer length of 20 nt exhibited the optimal activity. This may due to less *cis-*cleavage compared with 24 nt, and the accumulation of *cis-*cleavage products can inhibit *trans*-cleavage activity [6, 31]. To test the sequence universality of the Hp-structured activator, we selected three sequences commonly used in DNA mutation detection [32] (Fig. 2J). As shown in Fig. 2K, the activation efficiency of the Hp was always significantly higher than that of the Ds, while the activation efficiencies showed differences with different sequences.

### Reverse loop brings stronger activation effects

**Figure 3.**
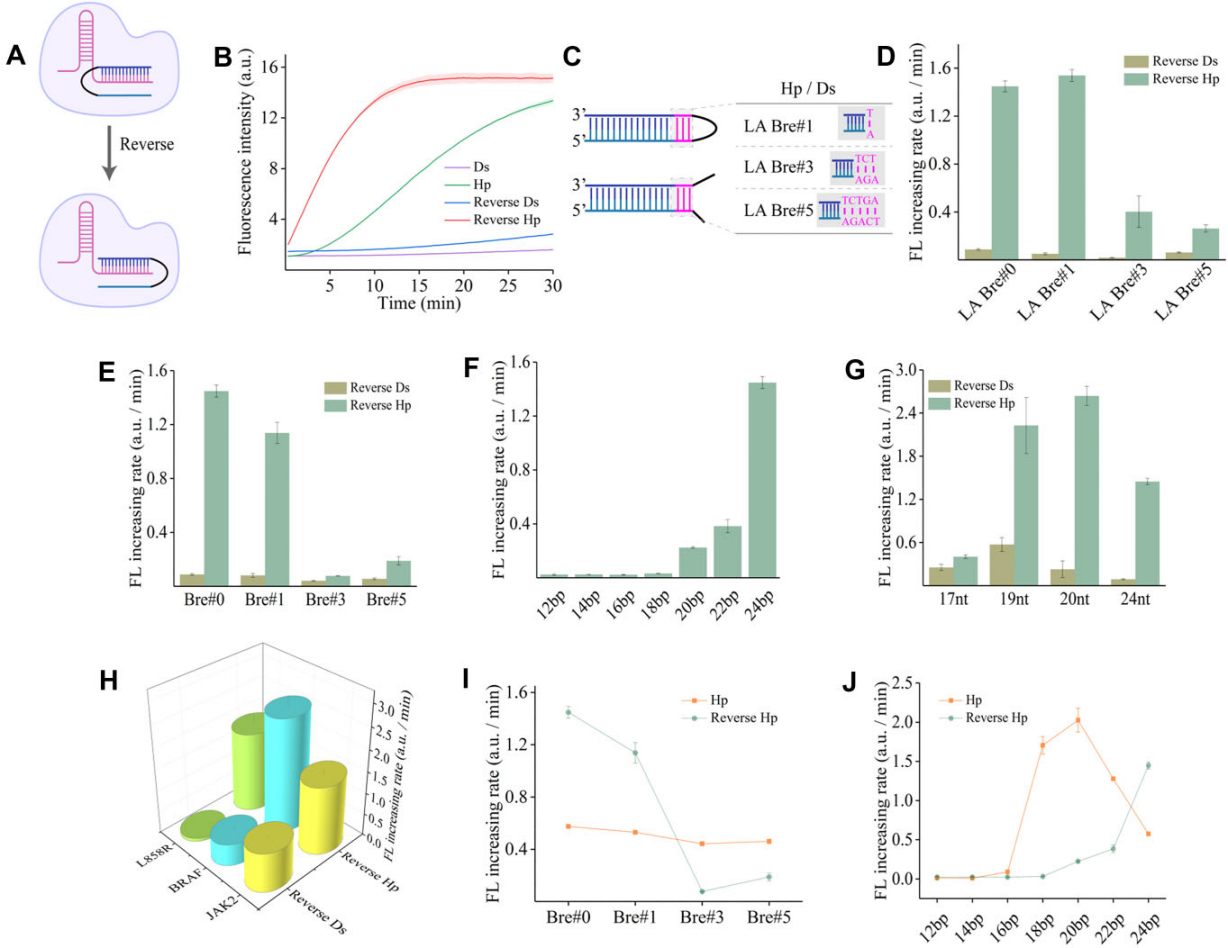
A Hp substrate with an inverted loop structure exhibits stronger *trans*-cleavage activity against Cas12a. (**A**) Schematic of a reverse Hp to activate *trans*-cleavage activity. (**B**) Feasibility of a reverse Hp for activation of Cas12a. (**C**) Schematic showing the positioning of the loop-adjacent extension of substrates. (**D**) Results of Hp and dsDNA substrate with different loop-adjacent extensions for Cas12a's *trans*-cleavage activity. The impact of (**E**) breathing dynamics, (**F**) stem length, (**G**) spacer length, and (**H**) spacer sequence on the activation of Cas12a by reverse Hp. Comparison of (**I**) ended-extension and (**J**) ended-shortening of the Hp-structured substrate on the activation of Cas12a between Hp and reverse Hp. Represented as the fluorescence increasing rate. Error bar, SD, *n* = 3.

We hypothesize that the tension brought by the loop structure (termed as the steric effect) facilitates the unwinding of the Hp stem. Consequently, we altered the position of the loop structure at the distal end of the protospacer to obtain the reverse Hp (Fig. 3A; Supplementary Fig. S4). The results indicated that reverse Hp can activate the *trans*-cleavage activity of Cas12a while showing a higher activation efficiency compared with Hp (Fig. 3B). We speculate that these differences arise from the position of the distal loop being closer to the *cis-*cleavage site, which has been verified to occur near 18 nt in TS and 22 nt in NTS. The inverted loop of the hairpin may facilitate the unwinding of the distal end, allowing it to complete *cis-*cleavage more favorably and initiate *trans*-cleavage, resulting in a faster fluorescence increasing rate. This observation also exists in other Cas12a orthologs (Supplementary Fig. S5). Additionally, molecular docking results showed that reverse Hp interacts with crRNA in RNP more than dsDNA without a PAM (Supplementary Fig. S6). Similarly, the loop-adjacent DNA breathing dynamics (LA-Bre) of the duplex also inhibited activation, further corroborating the role of the steric effect of the loop (Fig. 3C–D). To illustrate that the weakening of activation efficiency is not caused by the length of the activator, a corresponding comparison was also made with dsDNA activators containing the PAM, which did not cause any difference in the activation efficiency (Supplementary Fig. S7). Analogously, we explored the effect of breathing dynamics (Fig. 3E), stem length (Fig. 3F), spacer length (Fig. 3G), and spacer sequence (Fig. 3H) on *trans*-cleavage activity by reverse Hp. As expected, the activation efficiency of all reverse Hps was significantly higher than that of the reverse Ds. Unlike Hp, extending or shortening the stem of reverse Hp both notably reduce the activation efficiency, which may be attributed to the strict constraints of the seed region or the PAM region (Fig. 3I–J). The extension of the stem of the reverse Hp occupies the original PAM region of Cas12a RNP, and the shortening of this stem disrupts the binding area of the seed region. The successive shortening of Hp and reverse Hp from the stem near the loop structure (seed region for Hp and distant from seed region of reverse Hp) also indicates the stringency of the seed region and PAM region (Supplementary Fig. S8). Even the modification with a fluorescent dye at the terminus of reverse Hp significantly suppressed the activity (Supplementary Fig. S9), whereas an extension as long as 25 bp of Hp could still achieve a relatively high activation efficiency (Supplementary Fig. S10).

### Loop sequence and structure preference

**Figure 4.**
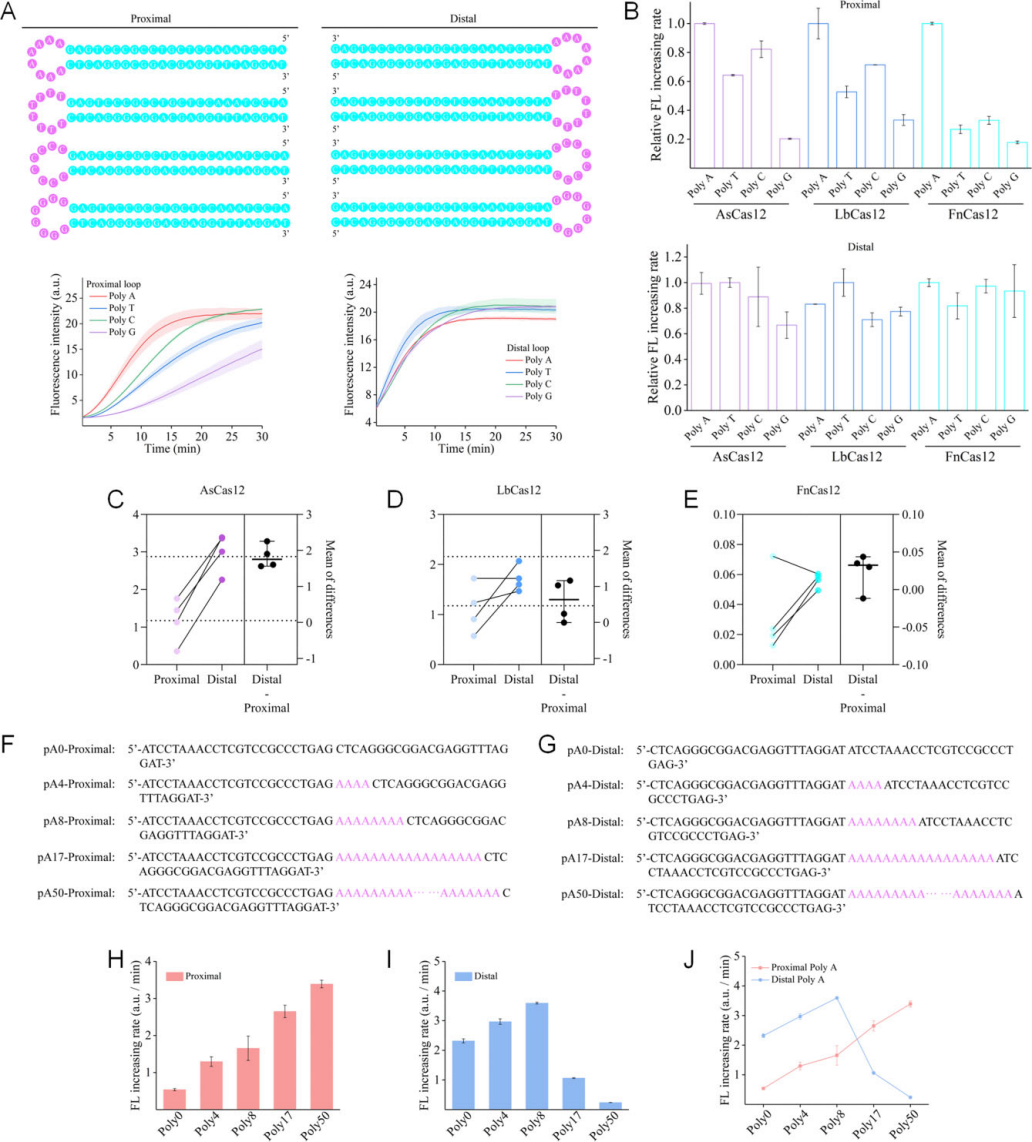
Analysis of loop sequence and structure preference. (**A**) Schematic of a Hp as proximal loop and reverse Hp as distal loop with an 8 nt poly-A/T/C/G sequence within the loop and the preference-caused difference of *trans*-cleavage activity of LbCas12a. (**B**) The activation differences of Cas12a orthologs activated by a proximal loop Hp and a distal loop Hp. Pairwise comparison of activation efficiency between proximal and distal loop Hp substrates on (**C**) AsCas12a, (**D**) LbCas12a, and (**E**) FnCas12a. Schematic of different loop sizes for (**F**) proximal and (**G**) distal poly-A loop Hp variants. (**H–J**) Results and comparison of the *trans*-cleavage activity of Cas12a activated by proximal and distal loop Hps with different loop sizes. Represented as the fluorescence increasing rate. Error bar, SD, *n* = 3.

To determine the sequence and structural requirements for the optimal *trans*-cleavage activity of Cas12a activated by Hp-structured activators, we investigated the sequence and structural preference of Cas12a in recognizing Hp-structured activators, examining the effects of both Hp and reverse Hp (termed proximal and distal Hps, according to the distance between the loop and the seed region) on loop sequences and sizes. The cleavage result demonstrated that the proximal loop Hp exhibits a more pronounced sequence preference, namely poly-A > poly-C > poly-T > poly-G, while there was essentially no difference in the distal loop Hp (Fig. 4A). This difference in preference was also observed in AsCas12a and FnCas12a (Fig. 4B; Supplementary Fig. S11). Comparing the activation efficiency of the proximal loop Hp and distal loop Hp among the Cas12a orthologs, the steric effect brought about by the distal loop is stronger than that by the proximal loop, and its activation efficiency is higher than that of the proximal loop Hp within the equivalent loop sequence and size (Fig. 4C–E). Subsequently, we selected poly-A to determine whether there is a loop size requirement for the Hp activator. Different poly-A loop sizes were designed and named pAx proximal or pAx distal, where x represents the size of the loop, namely 0, 4, 8, 17, and 50 nt (Fig. 4F–G). It should be noted that the interaction between the signal reporters (poly-T ssDNA) and the poly-A loop can be ignored (Supplementary Fig. S12). Interestingly, as the size of the loop increased, the *trans*-cleavage activity of Cas12a towards the proximal loop Hp increased (Fig. 4H), but the distal loop showed a trend of initially increasing and then decreasing after exceeding a loop size of 8 nt (Fig. 4J). It is possible that the oversized loop occupied the catalytic pocket of the RuvC domain responsible for *trans*-cleavage activity of Cas12a. This could impede the entry of the ssDNA reporter into the catalytic pocket and the subsequent non-specific cleavage, leading to a markedly reduced fluorescence increasing rate. The mechanisms involved need to be further explored in the future. In general, these findings are of great importance for the application of Cas12a in allosteric systems, especially in the design principles of Hp structure-related strategies, such as catalytic hairpin amplification (CHA) [33, 34] and hybridization chain reaction (HCR) [35, 36].

### Non-nucleic acid target detection with hairpin activators

The development of allosteric detection strategies for non-nucleic acid targets using Cas12a activators presents significant challenges, as these activators are often designed to bind with aptamer sequences specific to the molecular targets. Nevertheless, insufficient aptamer results in inadequate inhibition of the activator, while superfluous aptamer leads to depletion of the molecular targets, which are primary factors responsible for reduced sensitivity in non-nucleic acid target detection [37]. For other methods that do not rely on auxiliary molecules to inhibit activity, complex allosteric probe design [38] or time-consuming strand polymerization [39] are often required to achieve the conversion of non-nucleic acid targets into the components of the Cas12a system. By leveraging the distinct characteristics of PAM-free Hp and dsDNA activators, we developed a rapid and sensitive method for detecting non-nucleic acid targets based on a Hp-to-dsDNA (ON-to-OFF) allosteric strategy, which can effectively avoid background leakage and target loss without complex design.

**Figure 5.**
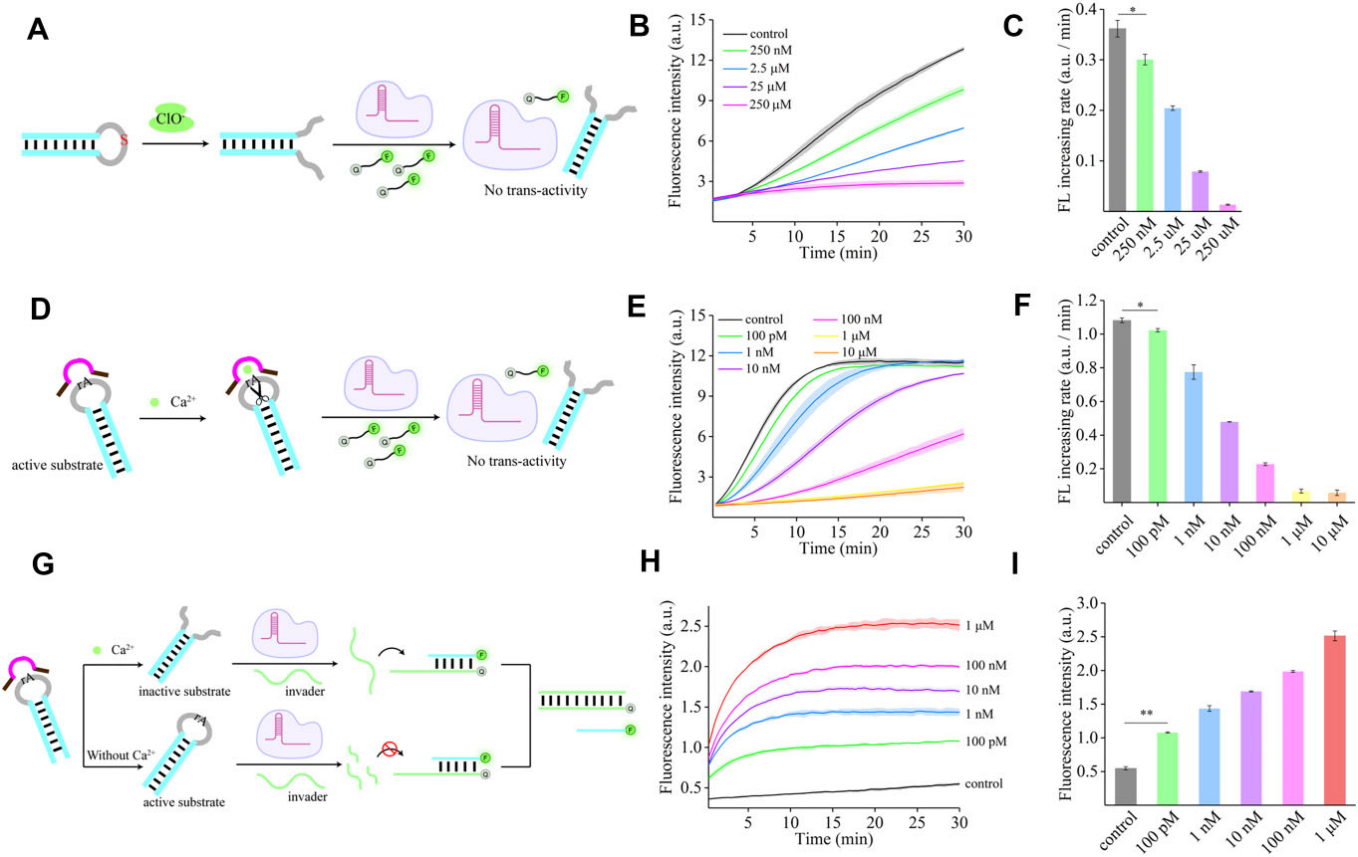
Specificity and sensitivity of Hp activator-mediated allosteric non-nucleic acid detection. (**A**) Schematic diagram of HOCl detection. (**B**) Fluorescence curves of an allosteric Hp substrate induced by different concentrations of HOCl. (**C**) Comparison of the fluorescence increasing rate from the fluorescence curves in (B). (**D**) Schematic diagram of calcium ion detection. (**E**) Fluorescence curves of allosteric Hp substrate induced by different concentrations of calcium ions. (**F**) Comparison of the fluorescence increasing rate from the fluorescence curves in (E). Hp activator-mediated allosteric non-nucleic acid detection in turn-on mode. (**G**) Schematic diagram of calcium ion detection. (**H**) Fluorescence curves of allosteric hairpin substrate induced by different concentrations of calcium ions. (**I**) Comparison of fluorescence intensity from the fluorescence curves in (H). Error bar, SD, *n* = 3. Data were analyzed by two-tailed t-test. *P< 0.05, **P< 0.01.

We investigated the sensitivity of this method using as a model target HOCl, which is one of the reactive oxygen species (ROS), and acts biologically as a marker for inflammatory and cardiovascular diseases, and so on [40, 41]. Specifically, we employed phosphorothioate modification in the loop of the Hp activator, which interacts with HOCl to facilitate the conformational transition from the active PAM-free Hp activators to inactive PAM-lacking dsDNA activators (Fig. 5A). Through exploration of different modification sites, we found that although phosphorothioate modification in the loop reduced activation efficiency, it still exhibited a significant difference compared with the converted dsDNA activator (Supplementary Fig. S13). As anticipated, the presence of HOCl resulted in a substantial decrease in fluorescence. Using this allosteric assay, HOCl at a concentration of 250 nM can be detected and effectively distinguished (Fig. 5B–C).

Besides the oxidation–reduction reaction, we constructed another allosteric approach based on DNAzyme for detection of metal ions. The ion-responsive DNAzyme consists of a catalytic center and recognition arms, and cleaves substrates containing adenine ribonucleotides (rAs) [42]. Herein, we used the loop of the Hp activators as DNAzyme cleavage substrates and selected EtNa DNAzyme to respond to calcium ions (Ca^2+^). In the presence of Ca^2+^, the catalytic center of DNAzyme can be activated and can cleave the loop of the Hp activator to achieve the transition from the active PAM-free Hp activators to inactive PAM-lacking dsDNA activators (Fig. 5D). Similarly, the fluorescence signal decreased with increasing concentration of Ca^2+^, and a concentration as low as 100 pM Ca^2+^ can be detected (Fig. 5E–F). For the selectivity analysis, only Ca^2+^ caused a specific fluorescence response, while Mg^2+^, Zn^2+^, Pb^2+^, Sr^2+^, and Mn^2+^ did not (Supplementary Fig. S14). In addition, recovery experiments were conducted in environmental water samples to verify its practical application. We tested the recovery rate for Ca^2+^ detection with environmental water samples (sourced from the Yangtze River, Supplementary Fig. S15). The results showed that the recovery rate ranged from 95% to 106.8%, indicating that this strategy is also applicable in complex real environmental samples (Supplementary Table S2). We have also developed a more suitable turn-on mode for sensing. Specifically, taking the detection of Ca^2+^ as an example, we replaced the original ssDNA reporter with a trigger strand that can initiate the replacement of the downstream dsDNA reporter. In the absence of Ca^2+^, the Hp-structured activator remains intact and can activate the *trans*-cleavage activity of Cas12a to cleave the trigger strand. Subsequently, when added to the dsDNA reporter, fluorescence signals cannot be obtained through strand displacement. In contrast, in the presence of Ca^2+^, Ca^2+^-dependent DNAzyme cleaves the Hp-structured activator, rendering it an inactive substrate of dsDNA lacking a PAM. This protects the trigger strand and enables the completion of the strand displacement reaction for the dsDNA reporter, achieving turn-on signal mode (Fig. 5G). In this mode, detection of Ca^2+^ as low as 100 pM can still be achieved (Fig. 5H–I). More importantly, the operability of DNAzyme allows for more biological applications to be connected upstream, providing multi-functionality for Hp activator-based Cas12a strategies. Upon comparison, the allosteric strategy we proposed exhibits an integrated advantage in terms of sensitivity, rapidity, and simplicity (Supplementary Table S3).

## Discussion

In the past, PAM-based active switches were mainly divided into several categories. One approach involves the introduction of a PAM through amplification methods such as RPA using a primer [43]. Another approach entails the formation of a PAM through hybridization via strand migration [27]. The new Hp-structured substrate allows us to design a stimulus-responsive allosteric probe by the flexibility of the loop region in molecular engineering. This represents a departure from the conventional PAM formation-based switches, introducing an alternative mode of switching based on conformational changes, which can serve as a new viable biosensing strategy.

**Figure 6.**
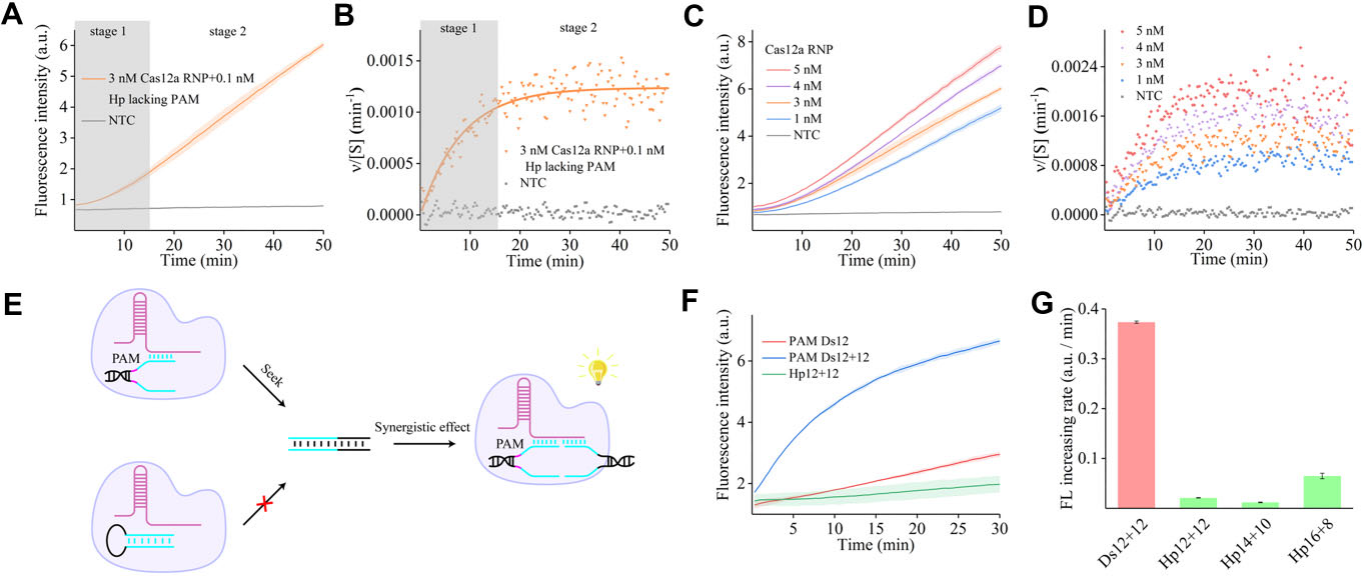
Determination of the dissociation constant and exploration of the synergistic effect of LbCas12a binding to the Hp-structured activator. (**A**) The real-time fluorescence curve of the cleaved reporter after the combination of RNP and Hp activator: non-linear phase of stage 1 and linear phase of stage 2. (**B**) The activator binding and LbCas12a activation kinetics. (**C**) The real-time fluorescence curve of the cleaved reporter after the combination of RNP and different concentrations of Hp activator. (**D**) The activator binding and LbCas12a activation kinetics measured by different concentrations of RNP. (**E**) Schematic of synergistic activation for Cas12a, dsDNA with PAM, and dsDNA synergistic activation shown above, and Hp without a PAM and dsDNA synergistic activation shown below. (**F**) The real-time fluorescence curve of synergistic activation by Ds12 + 12 and Hp12 + 12. (**G**) Comparison of synergistic activation efficiency after extending the length of the Hp substrate. Represented as the fluorescence increasing rate. Error bar, SD, *n* = 3.

To enhance our understanding of the proposed PAM-free Hp-structured activator for the *trans*-cleavage activity of Cas12a, we quantified the binding kinetics between the Cas12a RNP and the Hp activator. Referring to the method used by Feng *et al.* to calculate the kinetics of Cas13a with RNA [44], we monitored the real-time *trans*-cleavage activity to measure the real-time concentration [*E*] of the ternary complex formed between the Cas12a/crRNA RNP and the Hp activator. Taking the proximal loop Hp substrate as an example, a high excess [*RNP*] made the reaction rate determined solely by [*Hp*], and the reaction rate of the binding reaction for the Hp activator to RNP (Equation 1) can be simplified to follow pseudo-first-order kinetics (Equation 2). The initial non-linear phase was influenced by both binding kinetics and *trans*-cleavage reaction kinetics (stage 1 in Fig. 6A). After the reaction reached equilibrium, the linear phase reflected the sole control of the *trans*-cleavage reaction kinetics (stage 2 in Fig. 6A). Controlling the concentration of ssDNA reporter substrate [*S*]_0_ much lower than the Michaelis–Menten constant (*K*_m_ ≈ 725 nM), [*E*] was directly proportional to the real-time rate *ν*, and inversely proportional to the remaining substrate [*S*] (Equation 3). Furthermore, fitting *ν*/[*S*] versus *t* to an exponential function can give the apparent rate constant of binding (*k*_obs_) (Equation 4; Fig. 6B). By fitting the *k*_obs_ values of a series of different [*RNP*]_0_ to a linear equation of *k*_obs_ versus [*RNP*]_0_, the slope was *k*_2_ (*k*_on_) and the intercept was *k*_–2_ (*k*_off_), thereby giving the dissociation constant (*K*_d_) (Equation 5; Fig. 6C–D). The calculated *k*_on_, *k*_off_, and *K*_d_ for the Hp activator were 9.87 × 10^−3^/min/nM, 8.661 × 10^−2^/min, and 8.78 nM (Supplementary Fig. S16). Similarly, we also calculated the *K*_d_ between the PAM-lacking dsDNA activator and Cas12a, which was 103.48 nM (Supplementary Fig. S17). These results confirmed that the binding affinity of the Hp activator was higher than that of the PAM-lacking dsDNA activator. The detailed derivation process is provided in the Supplementary Information.


(1) \begin{eqnarray*}
RNP + activator\overset {{{k}_2}/{{k}_{ - 2}}} \longleftrightarrow E
\end{eqnarray*}

(2) \begin{eqnarray*}
[E] = {{[activator]}_0} - [activator]
\end{eqnarray*}

(3) \begin{eqnarray*}
E = \frac{{{{K}_M}\nu }}{{{{k}_{cat}}[S]}}
\end{eqnarray*}

(4) \begin{eqnarray*}
\frac{\nu }{{[S]}} = a \times {{e}^{ - \left( {{{k}_{obs}}} \right)t}} + b
\end{eqnarray*}

(5) \begin{eqnarray*}
{{K}_d} = \frac{{{{k}_{off}}}}{{{{k}_{on}}}} = \frac{{{{k}_{ - 2}}}}{{{{k}_2}}}
\end{eqnarray*}

The steric effect of the loop structure confers a relatively more effective activation on the Hp activator, and this steric effect is closely related to the parameters of the loop, including size, sequence, and position relative to the protospacer region. This steric effect substitutes for the inductive effect of the PAM, making the protospacer duplex more prone to unwinding, thereby allowing the TS to bind with the crRNA to form a DNA/RNA heteroduplex, and activating the *trans*-cleavage activity. However, such a substitution results in partial defects. In split activators, short dsDNA containing a PAM that inactivates the *trans*-cleavage can bind with the Cas12a RNP to capture another short dsDNA activator, and remotely induce the unwinding of another duplex, achieving synergistic activation (termed as exon-unwinding and induced targeting effect) [14]. Nevertheless, a short Hp activator cannot possess such properties, compared with the combination of a short 12 bp PAM-containing dsDNA and a 12 bp split dsDNA activator; even a 16 bp short Hp cannot activate Cas12a together with an 8 bp split dsDNA activator (Fig. 6E–G), suggesting that this steric effect also requires the assistance of the RNP. Because the short ssDNA activator at the proximal protospacer region also has exon-unwinding and induced targeting effect, the Hp with steric effects should theoretically activate the *trans*-cleavage activity of Cas12a after unwinding into a ssDNA, collaborating with another half activator. Only Hps that meet the activation length criteria show this steric effect, similar to how Cas12a provides lysine for PAM-containing duplexes that meet the requirements.

In addition, building upon our previous research [45], we attempted to induce steric allosteric changes in the loop structure of the Hp by leveraging the binding forces between nucleic acids and nucleic acids, and between small molecules and aptamers. The results indicated that neither the interaction between nucleic acids and nucleic acids nor that between small molecules and aptamers could restore the activity of the distal loop Hp activator, which was inhibited by the loop with a large size in the Cas12a system (Supplementary Fig. S18), i.e. the steric effect outweighs the binding effect, which may be related to the recognition process of the hairpin activator by Cas12a. It can be reasonably hypothesized that the Cas12a RNP recognizes the activator along the strand rather than directly targeting the loop structure. In the future, further investigation into the mechanism of the Hp activator is still required to expand its application in a broader range of Cas12a-based biological applications.

## Conclusion

The current substrates for Cas12a are limited to dsDNA containing a PAM or ssDNA. With the increasing demand for biological functionality, there is a growing need for more versatile substrates that can adapt to diverse scenarios. The restriction imposed by the PAM remains one of the primary challenges in the current Cas12a system. Although PAM-independent strategies have emerged, they rely on strand displacement to ultimately achieve activation by ssDNA substrates, which contradicts the ultimate intention of PAM-free activation. In this work, we demonstrated that a Hp-structured activator can elicit the *trans*-cleavage activity of Cas12a in a PAM-independent manner, which endows the Cas12a system with enhanced flexibility and scalability. By engineering the novel Hp activator, we constructed Hp-to-dsDNA allosteric strategies for the specific detection of non-nucleic acid targets, HOCl, and Ca^2+^, demonstrating satisfactory sensitivity. This finding will further advance the application of the Cas12a system and provide guidance for existing strategies.

## Supplementary Material

gkaf596_Supplemental_File

## Data Availability

The data underlying this article are available in the article and in its online supplementary material.

## Supplementary data

Supplementary data are available at NAR online.
