# Correction to ‘PAM-free hairpin target binding activates trans-cleavage activity of Cas12a’

**DOI:** 10.1093/nar/gkaf680

**Published:** 2025-07-16

This is a correction to: Xiaolong Li, Zixuan Zhu, Jiani Wu, Changjiang Li, Zhujun Liu, Jinjin Wang, Pu Li, Zhen Zhang, Yongming Huang, Jiaxin Hong, Tongbo Wu, PAM-free hairpin target binding activates trans-cleavage activity of Cas12a, Nucleic Acids Research, Volume 53, Issue 12, 8 July 2025, gkaf596, https://doi.org/10.1093/nar/gkaf596

The authors have noticed that the horizontal axes of subfigures H-J in Fig. 4 have been cropped and are not visible. Therefore, Fig. 4 has been replaced with an updated version.

This change does not affect the results, discussion and conclusions presented in the article. The published article has been updated.

