# Supplementary material for: PAM-free hairpin target binding activates trans-cleavage activity of Cas12a
Source: Nucleic Acids Res. 2025 Jun 25;53(12):gkaf596. doi: 10.1093/nar/gkaf596 (PMC12188295; doi:10.1093/nar/gkaf596)
Supplement: gkaf596_Supplemental_File [file gkaf596_supplemental_file.docx]

Supplementary Information

**PAM-free hairpin target binding activates trans-cleavage activity of Cas12a**

Xiaolong Li^1^, Zixuan Zhu^1^, Jiani Wu^1^, Changjiang Li^1^, Zhujun Liu^1^, Jinjin Wang^1^, Pu Li^1^, Zhen Zhang^2^, Yongming Huang^3^,*, Jiaxin Hong^4^,* and Tongbo Wu^1^,*

^1^ School of Pharmacy, Tongji Medical College, Huazhong University of Science and Technology, Wuhan, 430030, China

^2^ The First Affiliated Hospital, and College of Clinical Medicine of Henan University of Science and Technology, Luoyang, 471003, China

^3^ Department of Gastrointestinal Surgery, Union Hospital, Tongji Medical College, Huazhong University of Science and Technology, Wuhan, 430022, China

^4^ Cancer Center, Union Hospital, Tongji Medical College, Huazhong University of Science and Technology, Wuhan, 430022, China; Institute of Radiation Oncology, Union Hospital, Tongji Medical College, Huazhong University of Science and Technology, Wuhan, 430022, China; Hubei Province Key Laboratory of Precision Radiation Oncology, Wuhan, 430022, China

* To whom correspondence should be addressed. Tel: +86 27 83692754 (T. Wu); Fax: +86 27 83692754 (T. Wu); Email: [huangym90@outlook.com](mailto:huangym90@outlook.com) (Y. Huang), [hongesp91@outlook.com](mailto:hongesp91@outlook.com) (J. Hong), [wutongbo@hust.edu.cn](mailto:wutongbo@hust.edu.cn) (T. Wu)

**Kinetics of activation and trans-cleavage of Cas12a RNP with activator**

*(i) Formula derivation process*

If the formation of the ribonucleoprotein particle (RNP) reaches equilibrium prior to mixing with the activator, the apparent binding kinetics represent the binding step between the RNP and the activator. To ensure that the RNP reaches equilibrium, we pre-incubated Cas12a with crRNA for 30 minutes. Therefore, the binding reaction is considered solely as the association between the Cas12a RNP and the activator:

According to the rate law, the rate of this binding reaction is given by:

$$v_{2}=-\frac{d[activator]}{dt}=k_{2}[RNP][activator]-k_{-2}[E]$$

In the experiments, the concentration of Cas12a RNP was at least 10 times higher than that of the activator (from 1 nM to 5 nM RNP for 0.1 nM hairpin activator, and from 5 nm to 25 nM RNP for 0.1 nM dsDNA activator), satisfying the pseudo-first-order reaction conditions. Under these conditions, the reaction rate equation can be simplified to:

$$-\frac{d[activator]}{dt}=k_{2}'[activator]-k_{-2}[E]$$

Where $k_{2}'=k_{2}[RNP]_{0}$

$$-\frac{d[activator]}{dt}=k_{2}'[activator]-k_{-2}\left([activator]_{0}-[activator]\right)$$

According to the integrated rate law, the concentration of activator and ternary complex varies with time t as follows:

$$[E]=[activator]_{0}-[activator]=-[activator]_{0}\frac{k_{2}'}{k_{2}'+k_{-2}}e^{-\left(k_{2}'+k_{-2}\right)t}+[activator]_{0}\frac{k_{2}'}{k_{2}'+k_{-2}}$$

Based on Michaelis-Menten kinetics, where ν represents the cleavage rate of the reporter, ν_max_ denotes the maximum rate achievable at a given enzyme concentration and saturating substrate conditions. The catalytic rate constant or turnover number of the enzyme is represented by k_cat_, while K_M_ is the Michaelis constant. Given that the experimental conditions satisfy [E]«[S]«[K_M_] (the maximum concentration of the ternary complex is equivalent to the activator concentration of 0.1 nM, the reporter substrate concentration is 100 nM, and the reported K_M_ of LbCas12a is approximately 725 nM), the equation can be simplified, where [E] is directly proportional to the real-time rate ν and inversely proportional to the remaining substrate [S].

$$\nu=\frac{\nu_{\max}[S]}{K_{M}+[S]}=\frac{k_{cat}[E][S]}{K_{M}+[S]}$$

$$[E]=\frac{K_{M}\nu}{k_{cat}[S]}$$

Based on the aforementioned equations, fitting the real-time measurements of ν/[S] with the following function yields the apparent association rate constant (k_obs_) for the binding of RNP to the activator, where $k_{obs}=k_{2}'+k_{-2}$.

$$\frac{\nu}{[S]}=a\times e^{-\left(k_{obs}\right)t}+b$$

By performing a linear fit of the obtained k_obs_ values against the initial concentration of RNP ([RNP]_0_), the rate constants k_2_ (slope) and k_−2_ (intercept) are determined. Utilizing the following equation, the dissociation constant (K_d_) of the RNP-activator complex is derived based on the reaction rate constants k_2_ and k_−2_:

$$K_{d}=\frac{k_{-2}}{k_{2}}$$

*(ii) experimental procedure*

To determine the binding and dissociation rate constants of LbCas12a RNP with the activator, we employed a hairpin activator at a concentration of 0.1 nM and RNPs at concentrations of 1, 3, 4, and 5 nM. The concentration of LbCas12a was maintained at 50 nM, while the reporter concentration was set at 100 nM. The RNP was first incubated at 37°C for 30 minutes in NEBuffer r2.1, followed by mixing with the activator and reporter, and deionized water was added to bring the total volume to 20 µL. Real-time fluorescence was measured immediately after mixing, and the fluorescence signal was converted into the concentration of cleaved reporter based on the real-time fluorescence profile. A calibration curve was prepared by measuring the fluorescence generated from the complete cleavage of the reporter at different concentrations. The real-time cleavage rate was obtained between every two consecutive measurement points (measured every 20 seconds) using the Slope function. Fitting the plot of ν/[S] vs t to aforementioned exponential function to complete the calculation. Given the relatively low activation efficiency of LbCas12a by dsDNA without PAM, we used a dsDNA activator at 0.1 nM and RNPs at concentrations of 5, 15, 20, and 25 nM. The concentration of LbCas12a was kept at 50 nM, and the reporter concentration was 100 nM.

Table S1. Detailed sequences used in experiment.

| Name | Sequences from 5' to 3' |
| --- | --- |
| **Different activators for Cas12a's trans-cleavage** | |
| Reporter | FAM-TTTTTTTTTTTTTTT-BHQ-1 |
| Hp#1 | ATCCTAAACCTCGTCCGCCCTGAGCAAATTGCTCAGGGCGGACGAGGTTTAGGAT |
| Hp#2 | ATCCTAAACCTCGTCCGCCCTGAGCAAATGCTCAGGGCGGACGAGGTTTAGGAT |
| Hp#3 | ATCCTAAACCTCGTCCGCCCTGAGCAAAGCTCAGGGCGGACGAGGTTTAGGAT |
| Hp#4 | ATCCTAAACCTCGTCCGCCCTGAGCAAACTCAGGGCGGACGAGGTTTAGGAT |
| Hp#8 | ATCCTAAACCTCGTCCGCCCTGAGCAAAGGATCTCAGGGCGGACGAGGTTTAGGAT |
| Hp#17 | ATCCTAAACCTCGTCCGCCCTGAGCAAATGGAAAAAAGGATCTCAGGGCGGACGAGGTTTAGGAT |
| Ds-TS | ATCCTAAACCTCGTCCGCCCTGAG CAAA |
| Ds#1-NTS | TTA CTCAGGGCGGACGAGGTTTAGGAT |
| Ds#2-NTS | TA CTCAGGGCGGACGAGGTTTAGGAT |
| Ds#3-NTS | A CTCAGGGCGGACGAGGTTTAGGAT |
| Ds#4-NTS | CTCAGGGCGGACGAGGTTTAGGAT |
| Ds#8-NTS | GGAT CTCAGGGCGGACGAGGTTTAGGAT |
| Ds#17-NTS | TGGAAAAAAGGAT CTCAGGGCGGACGAGGTTTAGGAT |
| Hp Bre#0 | ATCCTAAACCTCGTCCGCCCTGAGCAAAGGATCTCAGGGCGGACGAGGTTTAGGAT |
| Hp Bre#1 | AATCCTAAACCTCGTCCGCCCTGAGCAAAGGATCTCAGGGCGGACGAGGTTTAGGATT |
| Hp Bre#3 | AGTATCCTAAACCTCGTCCGCCCTGAGCAAAGGATCTCAGGGCGGACGAGGTTTAGGATACT |
| Hp Bre#5 | AGTCTATCCTAAACCTCGTCCGCCCTGAGCAAAGGATCTCAGGGCGGACGAGGTTTAGGATAGACT |
| Ds Bre#0-TS | ATCCTAAACCTCGTCCGCCCTGAG CAAA |
| Ds Bre#0-NTS | GGAT CTCAGGGCGGACGAGGTTTAGGAT |
| Ds Bre#1-TS | A ATCCTAAACCTCGTCCGCCCTGAG CAAA |
| Ds Bre#1-NTS | GGAT CTCAGGGCGGACGAGGTTTAGGAT T |
| Ds Bre#3-TS | AGT ATCCTAAACCTCGTCCGCCCTGAG CAAA |
| Ds Bre#3-NTS | GGAT CTCAGGGCGGACGAGGTTTAGGAT ACT |
| Ds Bre#5-TS | AGTCT ATCCTAAACCTCGTCCGCCCTGAG CAAA |
| Ds Bre#5-NTS | GGAT CTCAGGGCGGACGAGGTTTAGGAT AGACT |
| Stem 12bp | GTCCGCCCTGAGCAAAGGATCTCAGGGCGGAC |
| Stem 14bp | TCGTCCGCCCTGAGCAAAGGATCTCAGGGCGGACGA |
| Stem 16bp | CCTCGTCCGCCCTGAGCAAAGGATCTCAGGGCGGACGAGG |
| Stem 18bp | AACCTCGTCCGCCCTGAG CAAAGGAT CTCAGGGCGGACGAGGTT |
| Stem 20bp | TAAACCTCGTCCGCCCTGAGCAAAGGATCTCAGGGCGGACGAGGTTTA |
| Stem 22bp | CCTAAACCTCGTCCGCCCTGAGCAAAGGATCTCAGGGCGGACGAGGTTTAGG |
| Stem 24bp | ATCCTAAACCTCGTCCGCCCTGAGCAAAGGATCTCAGGGCGGACGAGGTTTAGGAT |
| 17-nt spacer crRNA | UAAUUUCUACUAAGUGUAGAUCUCAGGGCGGACGAGGU |
| Hp 17nt | ACCTCGTCCGCCCTGAGCAAAGGATCTCAGGGCGGACGAGGT |
| Ds 17nt-TS | ACCTCGTCCGCCCTGAG CAAA |
| Ds 17nt-NTS | GGAT CTCAGGGCGGACGAGGT |
| 19-nt spacer crRNA | UAAUUUCUACUAAGUGUAGAUCUCAGGGCGGACGAGGUUU |
| Hp 19nt | AAACCTCGTCCGCCCTGAGCAAAGGATCTCAGGGCGGACGAGGTTT |
| Ds 19nt-TS | AAACCTCGTCCGCCCTGAG CAAA |
| Ds 19nt-NTS | GGAT CTCAGGGCGGACGAGGTTT |
| 20-nt spacer crRNA | UAAUUUCUACUAAGUGUAGAUCUCAGGGCGGACGAGGUUUA |
| Hp 20nt | TAAACCTCGTCCGCCCTGAGCAAAGGATCTCAGGGCGGACGAGGTTTA |
| Ds 20nt-TS | TAAACCTCGTCCGCCCTGAG CAAA |
| Ds 20nt-NTS | GGAT CTCAGGGCGGACGAGGTTTA |
| 24-nt spacer crRNA | UAAUUUCUACUAAGUGUAGAUCUCAGGGCGGACGAGGUUUAGGAU |
| Hp 24nt | ATCCTAAACCTCGTCCGCCCTGAGCAAAGGATCTCAGGGCGGACGAGGTTTAGGAT |
| Ds 24nt-TS | ATCCTAAACCTCGTCCGCCCTGAG CAAA |
| Ds 24nt-NTS | GGAT CTCAGGGCGGACGAGGTTTAGGAT |
| JAK2 crRNA | UAAUUUCUACUAAGUGUAGAUGAAACAUACUCCAUAAUUU |
| JAK2 Hp | AAATTATGGAGTATGTTTCCAAAGGATGAAACATACTCCATAATTT |
| JAK2 Ds-TS | AAATTATGGAGTATGTTTC CAAA |
| JAK2 Ds-NTS | GGAT GAAACATACTCCATAATTT |
| BRAF crRNA | UAAUUUCUACUAAGUGUAGAUUCUCUGUAGCUAGACCAAA |
| BRAF Hp | TTTGGTCTAGCTACAGAGACAAAGGATTCTCTGTAGCTAGACCAAA |
| BRAF Ds-TS | TTTGGTCTAGCTACAGAGA CAAA |
| BRAF Ds-NTS | GGAT TCTCTGTAGCTAGACCAAA |
| L858R crRNA | UAAUUUCUACUAAGUGUAGAUGCGGGCCAAACUGCUGGGU |
| L858R Hp | ACCCAGCAGTTTGGCCCGCCAAAGGATGCGGGCCAAACTGCTGGGT |
| L858R Ds-TS | ACCCAGCAGTTTGGCCCGC CAAA |
| L858R Ds-NTS | GGAT GCGGGCCAAACTGCTGGGT |
| Reverse Hp LA Bre#0 | CTCAGGGCGGACGAGGTTTAGGATTAGGAAACATCCTAAACCTCGTCCGCCCTGAG |
| Reverse Hp LA Bre#1 | CTCAGGGCGGACGAGGTTTAGGATATAGGAAACTATCCTAAACCTCGTCCGCCCTGAG |
| Reverse Hp LA Bre#3 | CTCAGGGCGGACGAGGTTTAGGATAGATAGGAAACTCTATCCTAAACCTCGTCCGCCCTGAG |
| Reverse Hp LA Bre#5 | CTCAGGGCGGACGAGGTTTAGGATAGACTTAGGAAACAGTCTATCCTAAACCTCGTCCGCCCTGAG |
| Reverse Ds LA Bre#0-TS | AAAC ATCCTAAACCTCGTCCGCCCTGAG |
| Reverse Ds LA Bre#0-NTS | CTCAGGGCGGACGAGGTTTAGGAT TAGG |
| Reverse Ds LA Bre#1-TS | AAAC TATCCTAAACCTCGTCCGCCCTGAG |
| Reverse Ds LA Bre#1-NTS | CTCAGGGCGGACGAGGTTTAGGATA TAGG |
| Reverse Ds LA Bre#3-TS | AAAC TCTATCCTAAACCTCGTCCGCCCTGAG |
| Reverse Ds LA Bre#3-NTS | CTCAGGGCGGACGAGGTTTAGGATAGA TAGG |
| Reverse Ds LA Bre#5-TS | AAAC AGTCTATCCTAAACCTCGTCCGCCCTGAG |
| Reverse Ds LA Bre#5-NTS | CTCAGGGCGGACGAGGTTTAGGATAGACT TAGG |
| Reverse Hp Bre#0 | CTCAGGGCGGACGAGGTTTAGGATTAGGAAACATCCTAAACCTCGTCCGCCCTGAG |
| Reverse Hp Bre#1 | TCTCAGGGCGGACGAGGTTTAGGATTAGGAAACATCCTAAACCTCGTCCGCCCTGAGA |
| Reverse Hp Bre#3 | TCACTCAGGGCGGACGAGGTTTAGGATTAGGAAACATCCTAAACCTCGTCCGCCCTGAGTGA |
| Reverse Hp Bre#5 | TCAGACTCAGGGCGGACGAGGTTTAGGATTAGGAAACATCCTAAACCTCGTCCGCCCTGAGTCTGA |
| Reverse Ds Bre#0-TS | AAAC ATCCTAAACCTCGTCCGCCCTGAG |
| Reverse Ds Bre#0-NTS | CTCAGGGCGGACGAGGTTTAGGAT TAGG |
| Reverse Ds Bre#1-TS | AAAC ATCCTAAACCTCGTCCGCCCTGAGA |
| Reverse Ds Bre#1-NTS | TCTCAGGGCGGACGAGGTTTAGGAT TAGG |
| Reverse Ds Bre#3-TS | AAAC ATCCTAAACCTCGTCCGCCCTGAGTGA |
| Reverse Ds Bre#3-NTS | TCACTCAGGGCGGACGAGGTTTAGGAT TAGG |
| Reverse Ds Bre#5-TS | AAAC ATCCTAAACCTCGTCCGCCCTGAGTCTGA |
| Reverse Ds Bre#5-NTS | TCAGACTCAGGGCGGACGAGGTTTAGGAT TAGG |
| Reverse Stem 12bp | GAGGTTTAGGATTAGGAAACATCCTAAACCTC |
| Reverse Stem 14bp | ACGAGGTTTAGGATTAGGAAACATCCTAAACCTCGT |
| Reverse Stem 16bp | GGACGAGGTTTAGGATTAGGAAACATCCTAAACCTCGTCC |
| Reverse Stem 18bp | GCGGACGAGGTTTAGGATTAGGAAACATCCTAAACCTCGTCCGC |
| Reverse Stem 20bp | GGGCGGACGAGGTTTAGGATTAGGAAACATCCTAAACCTCGTCCGCCC |
| Reverse Stem 22bp | CAGGGCGGACGAGGTTTAGGATTAGGAAACATCCTAAACCTCGTCCGCCCTG |
| Reverse Stem 24bp | CTCAGGGCGGACGAGGTTTAGGATTAGGAAACATCCTAAACCTCGTCCGCCCTGAG |
| 17-nt spacer crRNA | UAAUUUCUACUAAGUGUAGAUCUCAGGGCGGACGAGGU |
| Reverse Hp 17nt | CTCAGGGCGGACGAGGTTAGGAAACACCTCGTCCGCCCTGAG |
| Reverse Ds 17nt-TS | AAACACCTCGTCCGCCCTGAG |
| Reverse Ds 17nt-NTS | CTCAGGGCGGACGAGGTTAGG |
| 19-nt spacer crRNA | UAAUUUCUACUAAGUGUAGAUCUCAGGGCGGACGAGGUUU |
| Reverse Hp 19nt | CTCAGGGCGGACGAGGTTTTAGGAAACAAACCTCGTCCGCCCTGAG |
| Reverse Ds 19nt-TS | AAACAAACCTCGTCCGCCCTGAG |
| Reverse Ds 19nt-NTS | CTCAGGGCGGACGAGGTTTTAGG |
| 20-nt spacer crRNA | UAAUUUCUACUAAGUGUAGAUCUCAGGGCGGACGAGGUUUA |
| Reverse Hp 20nt | CTCAGGGCGGACGAGGTTTATAGGAAACTAAACCTCGTCCGCCCTGAG |
| Reverse Ds 20nt-TS | AAACTAAACCTCGTCCGCCCTGAG |
| Reverse Ds 20nt-NTS | CTCAGGGCGGACGAGGTTTATAGG |
| 24-nt spacer crRNA | UAAUUUCUACUAAGUGUAGAUCUCAGGGCGGACGAGGUUUAGGAU |
| Reverse Hp 24nt | CTCAGGGCGGACGAGGTTTAGGATTAGGAAACATCCTAAACCTCGTCCGCCCTGAG |
| Reverse Ds 24nt-TS | AAACATCCTAAACCTCGTCCGCCCTGAG |
| Reverse Ds 24nt-NTS | CTCAGGGCGGACGAGGTTTAGGATTAGG |
| Reverse JAK2 Hp | GAAACATACTCCATAATTTTAGGAAACAAATTATGGAGTATGTTTC |
| Reverse JAK2 Ds-TS | AAAC AAATTATGGAGTATGTTTC |
| Reverse JAK2 Ds-NTS | GAAACATACTCCATAATTT TAGG |
| Reverse BRAF Hp | TCTCTGTAGCTAGACCAAATAGGAAACTTTGGTCTAGCTACAGAGA |
| Reverse BRAF Ds-TS | AAAC TTTGGTCTAGCTACAGAGA |
| Reverse BRAF Ds-NTS | TCTCTGTAGCTAGACCAAA TAGG |
| Reverse L858R Hp | GCGGGCCAAACTGCTGGGTTAGGAAACACCCAGCAGTTTGGCCCGC |
| Reverse L858R Ds-TS | AAAC ACCCAGCAGTTTGGCCCGC |
| Reverse L858R Ds-NTS | GCGGGCCAAACTGCTGGGT TAGG |
| Hp LA shorten 12bp | ATCCTAAACCTCCAAAGGATGAGGTTTAGGAT |
| Hp LA shorten 14bp | ATCCTAAACCTCGTCAAAGGATACGAGGTTTAGGAT |
| Hp LA shorten 16bp | ATCCTAAACCTCGTCCCAAAGGATGGACGAGGTTTAGGAT |
| Hp LA shorten 18bp | ATCCTAAACCTCGTCCGCCAAAGGATGCGGACGAGGTTTAGGAT |
| Hp LA shorten 20bp | ATCCTAAACCTCGTCCGCCCCAAAGGATGGGCGGACGAGGTTTAGGAT |
| Hp LA shorten 22bp | ATCCTAAACCTCGTCCGCCCTGCAAAGGATCAGGGCGGACGAGGTTTAGGAT |
| Hp LA shorten 24bp | ATCCTAAACCTCGTCCGCCCTGAGCAAAGGATCTCAGGGCGGACGAGGTTTAGGAT |
| Reverse Hp LA shorten 12bp | CTCAGGGCGGACTAGGAAACGTCCGCCCTGAG |
| Reverse Hp LA shorten 14bp | CTCAGGGCGGACGATAGGAAACTCGTCCGCCCTGAG |
| Reverse Hp LA shorten 16bp | CTCAGGGCGGACGAGGTAGGAAACCCTCGTCCGCCCTGAG |
| Reverse Hp LA shorten 18bp | CTCAGGGCGGACGAGGTTTAGGAAACAACCTCGTCCGCCCTGAG |
| Reverse Hp LA shorten 20bp | CTCAGGGCGGACGAGGTTTATAGGAAACTAAACCTCGTCCGCCCTGAG |
| Reverse Hp LA shorten 22bp | CTCAGGGCGGACGAGGTTTAGGTAGGAAACCCTAAACCTCGTCCGCCCTGAG |
| Reverse Hp LA shorten 24bp | CTCAGGGCGGACGAGGTTTAGGATTAGGAAACATCCTAAACCTCGTCCGCCCTGAG |
| Reverse Hp-HEX,BHQ | HEX-CTCAGGGCGGACGAGGTTTAGGATTAGGAAACATCCTAAACCTCGTCCGCCCTGAG-BHQ-1 |
| Hp Bre#25 | AGTCTAGTCTAGTCTAGTCTAGTCTATCCTAAACCTCGTCCGCCCTGAGCAAAGGATCTCAGGGCGGACGAGGTTTAGGATAGACTAGACTAGACTAGACTAGACT |
| **Loop sequence and structure preference** | |
| Proximal Poly A | ATCCTAAACCTCGTCCGCCCTGAGAAAAAAAACTCAGGGCGGACGAGGTTTAGGAT |
| Proximal Poly T | ATCCTAAACCTCGTCCGCCCTGAGTTTTTTTTCTCAGGGCGGACGAGGTTTAGGAT |
| Proximal Poly C | ATCCTAAACCTCGTCCGCCCTGAGCCCCCCCCCTCAGGGCGGACGAGGTTTAGGAT |
| Proximal Poly G | ATCCTAAACCTCGTCCGCCCTGAGGGGGGGGGCTCAGGGCGGACGAGGTTTAGGAT |
| Distal Poly A | CTCAGGGCGGACGAGGTTTAGGATAAAAAAAAATCCTAAACCTCGTCCGCCCTGAG |
| Distal Poly T | CTCAGGGCGGACGAGGTTTAGGATTTTTTTTTATCCTAAACCTCGTCCGCCCTGAG |
| Distal Poly C | CTCAGGGCGGACGAGGTTTAGGATCCCCCCCCATCCTAAACCTCGTCCGCCCTGAG |
| Distal Poly G | CTCAGGGCGGACGAGGTTTAGGATGGGGGGGGATCCTAAACCTCGTCCGCCCTGAG |
| PA0-Proximal | ATCCTAAACCTCGTCCGCCCTGAGCTCAGGGCGGACGAGGTTTAGGAT |
| PA4-Proximal | ATCCTAAACCTCGTCCGCCCTGAGAAAACTCAGGGCGGACGAGGTTTAGGAT |
| PA8-Proximal | ATCCTAAACCTCGTCCGCCCTGAGAAAAAAAACTCAGGGCGGACGAGGTTTAGGAT |
| PA17-Proximal | ATCCTAAACCTCGTCCGCCCTGAGAAAAAAAAAAAAAAAAACTCAGGGCGGACGAGGTTTAGGAT |
| PA50-Proximal | ATCCTAAACCTCGTCCGCCCTGAGAAAAAAAAAAAAAAAAAAAAAAAAAAAAAAAAAAAAAAAAAAAAAAAAAACTCAGGGCGGACGAGGTTTAGGAT |
| PA0-Distal | CTCAGGGCGGACGAGGTTTAGGATATCCTAAACCTCGTCCGCCCTGAG |
| PA4-Distal | CTCAGGGCGGACGAGGTTTAGGATAAAAATCCTAAACCTCGTCCGCCCTGAG |
| PA8-Distal | CTCAGGGCGGACGAGGTTTAGGATAAAAAAAAATCCTAAACCTCGTCCGCCCTGAG |
| PA17-Distal | CTCAGGGCGGACGAGGTTTAGGATAAAAAAAAAAAAAAAAAATCCTAAACCTCGTCCGCCCTGAG |
| PA50-Distal | CTCAGGGCGGACGAGGTTTAGGATAAAAAAAAAAAAAAAAAAAAAAAAAAAAAAAAAAAAAAAAAAAAAAAAAAATCCTAAACCTCGTCCGCCCTGAG |
| **Non-nucleic acid targets detection** | |
| Thio-Reverse Hp-0 | CTCAGGGCGGACGAGGTTTAGGATTAGGAAAC*ATCCTAAACCTCGTCCGCCCTGAG |
| Thio-Reverse Hp-1 | CTCAGGGCGGACGAGGTTTAGGATTAGGAAA*CATCCTAAACCTCGTCCGCCCTGAG |
| Thio-Reverse Hp-2 | CTCAGGGCGGACGAGGTTTAGGATTAGGAA*ACATCCTAAACCTCGTCCGCCCTGAG |
| Thio-Reverse Hp-3 | CTCAGGGCGGACGAGGTTTAGGATTAGGA*AACATCCTAAACCTCGTCCGCCCTGAG |
| Thio-Reverse Hp-4 | CTCAGGGCGGACGAGGTTTAGGATTAGG*AAACATCCTAAACCTCGTCCGCCCTGAG |
| Ca^2+^-DNAzyme | GCCCATCTTTTCTCACACCGTACTCGGTAAGGTTGT  TAGTGA |
| DNAzyme-Hp | ATCCTAAACCTCGTCCGCCCTGAGTCACTAT/rA**/**GGAAGATGGGCCTCAGGGCGGACGAGGTTTAGGAT |
| **Discussion** | |
| PAM Ds12-TS | GTCCGCCCTGAGTAAAGCGA |
| PAM Ds12-NTS | TCGCTTTACTCAGGGCGGAC |
| Hp12 | GTCCGCCCTGAGCAAAGGATCTCAGGGCGGAC |
| Split Ds12-TS | TTGATAAAGCGACGCTGATAATCCTAAACCTC |
| Split Ds12-NTS | GAGGTTTAGGATTATCAGCGTCGCTTTATCAA |
| Hp14 | TCGTCCGCCCTGAGCAAAGGATCTCAGGGCGGACGA |
| Split Ds10-TS | TTGATAAAGCGACGCTGATAATCCTAAACC |
| Split Ds10-NTS | GGTTTAGGATTATCAGCGTCGCTTTATCAA |
| Hp16 | CCTCGTCCGCCCTGAGCAAAGGATCTCAGGGCGGACGAGG |
| Split Ds8-TS | TTGATAAAGCGACGCTGATAATCCTAAA |
| Split Ds8-NTS | TTTAGGATTATCAGCGTCGCTTTATCAA |
| Reverse Hp-ATP | CTCAGGGCGGACGAGGTTTAGGATAAAAGGGGGAGTATTGCGGAGGAGCGGGGGAGTATTGCGGAGGAAAAAATCCTAAACCTCGTCCGCCCTGAG |
| Reverse Hp-miR155 | CTCAGGGCGGACGAGGTTTAGGATAAAAATTAGCATTAAAAAAAAAAAAAAAAAAAAAAAAAAAAAAAAAAAAAAAAAAAAAACCCCTATCACGAAAAATCCTAAACCTCGTCCGCCCTGAG |
| miR155 | UUAAUGCUAAUCGUGAUAGGGGU |

Pink represents loop sequence, * represents phosphorothioate modification, underline represents the DNAzyme recognition arm region.

Table S2. Recovery experiments for Ca^2+^ in environmental water samples (n=6)

| Sample | Added | Found (mean ± SD) | Recovery (%) |
| --- | --- | --- | --- |
| 1 | 5 nM | 4.75 ± 0.2 nM | 95 |
| 2 | 50 nM | 47.87 ± 6.9 nM | 95.7 |
| 3 | 500 nM | 533.91 ± 37.8 nM | 106.8 |

Table S3. Comparison of different detection methods for detecting HOCl and Ca2+.

| Target | Detection strategy | Detection limit | Material prepare | Assay time | Reference |
| --- | --- | --- | --- | --- | --- |
| HOCl | CRISPR/Cas12-based RESET effect | 0.33 μM | 35 min | 1.7 h | [1] |
|  | Single wavelength laser excitation ratiometric NIR-II fluorescent probe | 0.4 μM | 13.5 h | unidentified | [2] |
|  | Cationic fluorescent carbon dots with solution ultra-stability | 0.32 μM | >36 h | 3 min | [3] |
|  | BI-PEG-PSBP NPs for fluorescent labeling strategy | 0.102 μM | >50 h | ≈10 min | [4] |
|  | Hairpin activator-mediated allosteric detection | 0.250 μM | 15 min | 1.5 h | This work |
| Ca^2+^ | Environmentally sensitive color-shifting fluorophores | 5 nM | >72 h | 15 min | [5] |
|  | Dopamine-modified  graphene quantum dots with dual emission mechanisms | 80 nM | >96 h | 5 min | [6] |
|  | DNAzyme-gold nanostar probe | 56 nM | 16.5 h | 30 min | [7] |
|  | Near-infrared nanosensor based on supramolecular self-assembly | 9.7 nM | >7 d | 30 min | [8] |
|  | Hairpin activator-mediated allosteric detection | 0.1 nM | 15 min | 1.5 h | This work |

More importantly, from a comprehensive perspective, it has more significant value, such as rapidity and simplicity. In practical sensing applications, sensitivity, rapidity, and simplicity are all considered as integral factors, which collectively facilitate the field-deployability of the sensor. From our probe preparation to target recognition and Cas12a signal output, the entire process can be completed in less than 2 hours. Compared with other strategies, although some studies have demonstrated superior sensitivity and advantages in detection time, these improvements are generally achieved at the expense of significantly increased material preparation time. In contrast, our method achieves a balanced trade-off between sensitivity, rapidity, and simplicity.


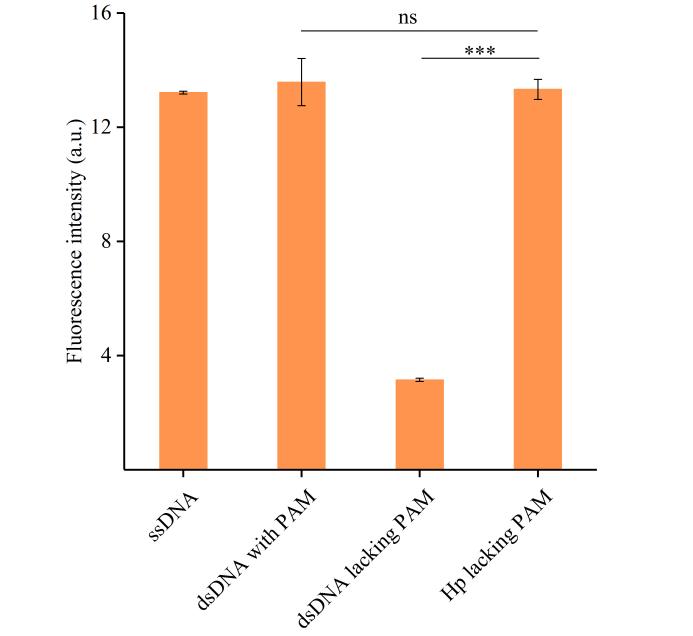


**Fig. S1.** Comparison of real-time fluorescence intensity of four types of activators at 30 minutes. Error bar, SD, n=3. Data were analyzed by two-tailed t-test. ns means no significance, ***p<0.001.


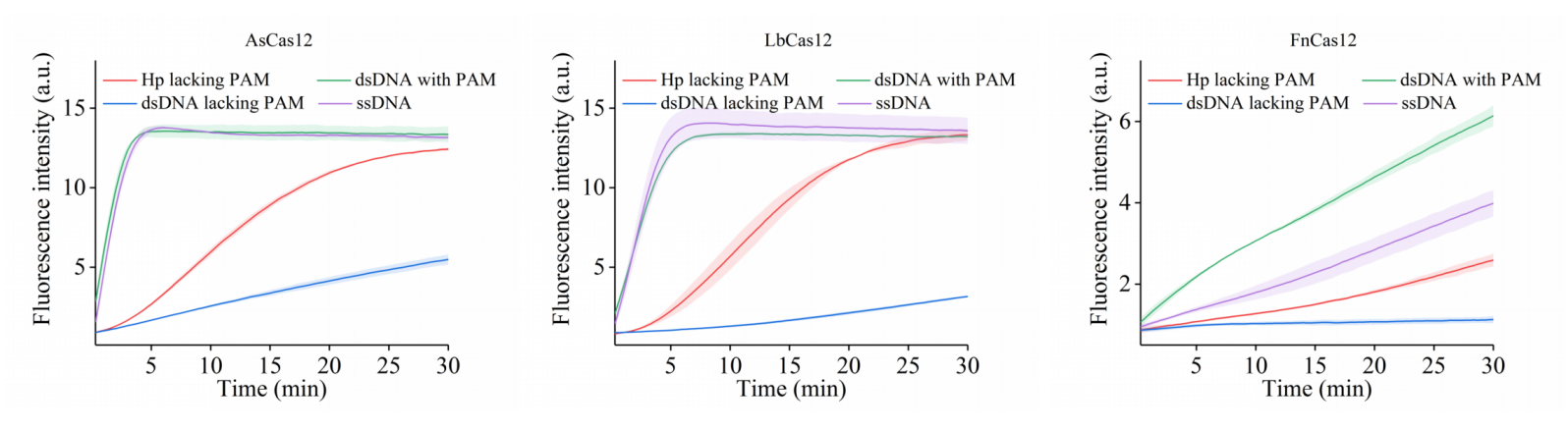


**Fig. S2.** The real-time fluorescence curves of the cleaved reporter for trans-cleavage cleavage of different Cas12a orthologs (AsCas12a, LbCas12a, FnCas12a) activated by four substrates of ssDNA, dsDNA with PAM, Hp lacking PAM, dsDNA lacking PAM. Error bar, SD, n=3.


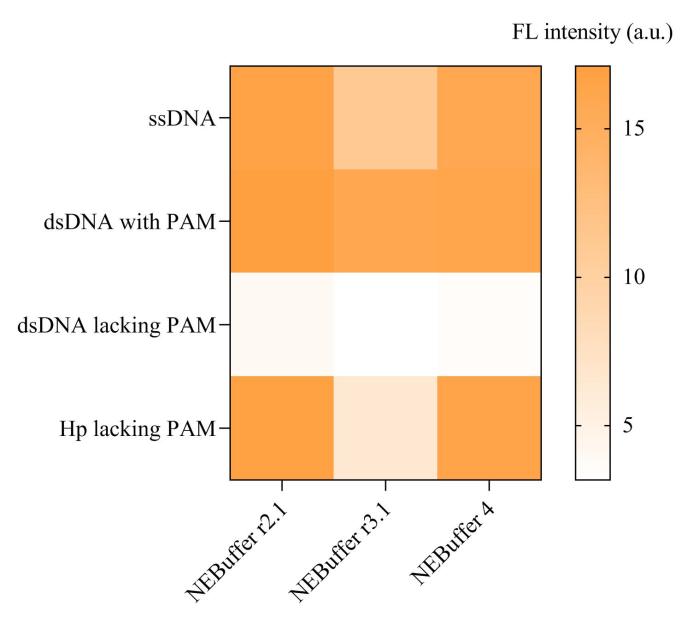


**Fig. S3.** The activation differences of four types of substrates in different reaction buffer, compared based on the fluorescence intensity of the cleaved reporter at 30 minutes.


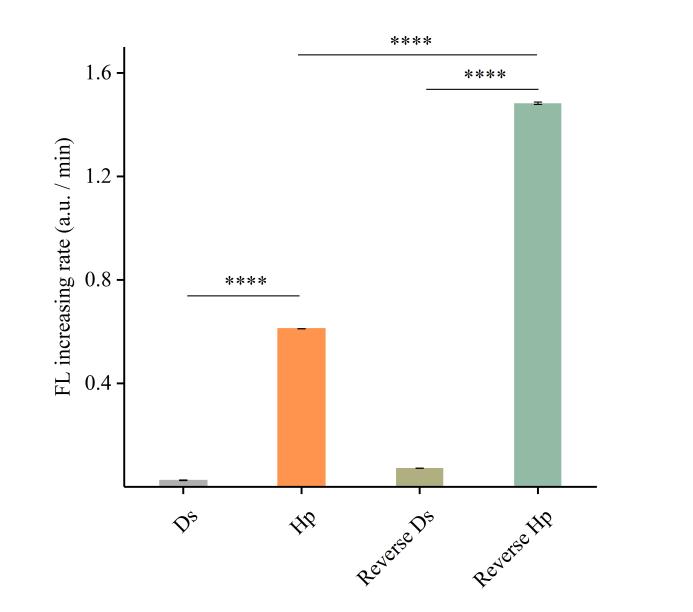


**Fig. S4.** Comparison of hairpin-structured substrate on the activation of Cas12a between hairpin and reverse hairpin. Error bar, SD, n=3. Data were analyzed by two-tailed t-test. ****p<0.0001.


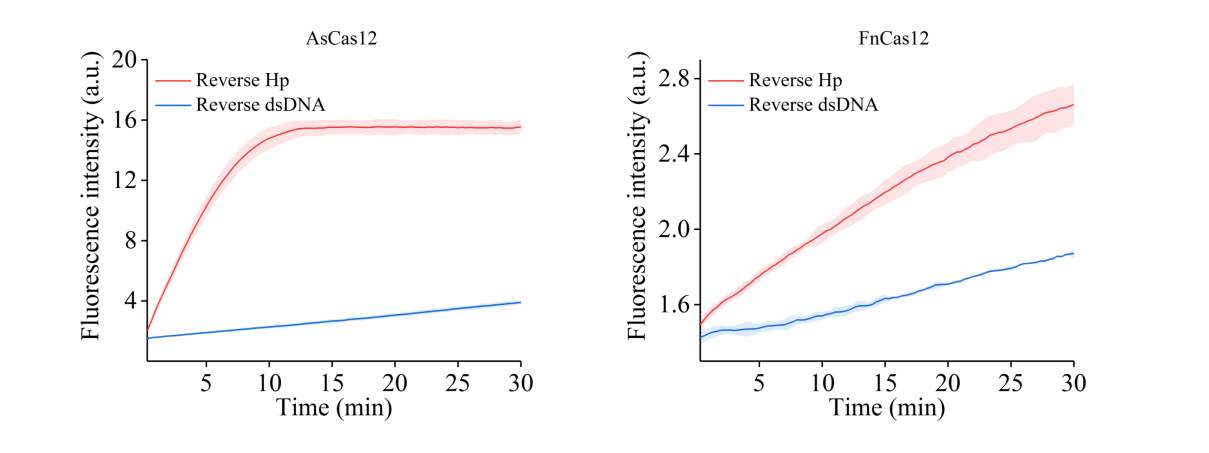


**Fig. S5.** The real-time fluorescence curves of the cleaved reporter for trans-cleavage cleavage of different Cas12a orthologs (AsCas12a, FnCas12a) activated by reverse hairpin. The activation result of LbCas12a is shown in Fig. 3B. Error bar, SD, n=3.


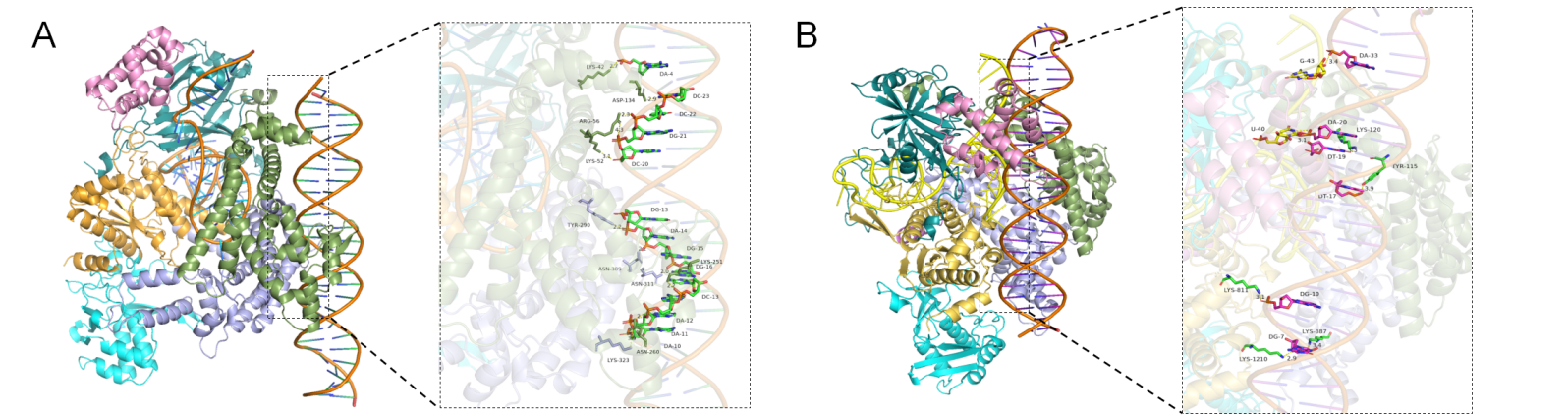


**Fig. S6.** (A) Molecular docking between reverse dsDNA lacking PAM substrate and Cas12a/crRNA RNP. (B) Molecular docking between reverse hairpin lacking PAM substrate and Cas12a/crRNA RNP.


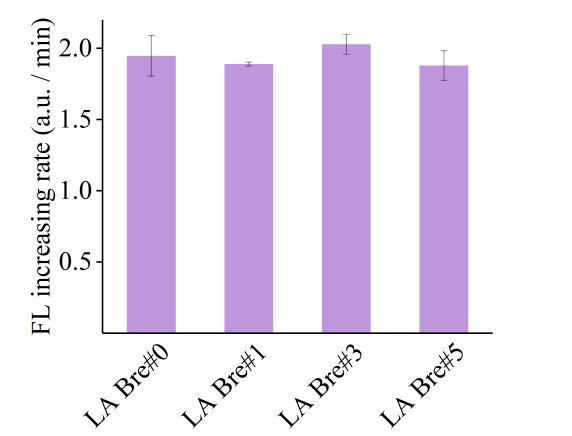


**Fig. S7.** Results of dsDNA containing PAM corresponding different loop adjacent extensions for Cas12a's trans-cleavage activity. Represented as fluorescence increasing rate, Error bar, SD, n=3.


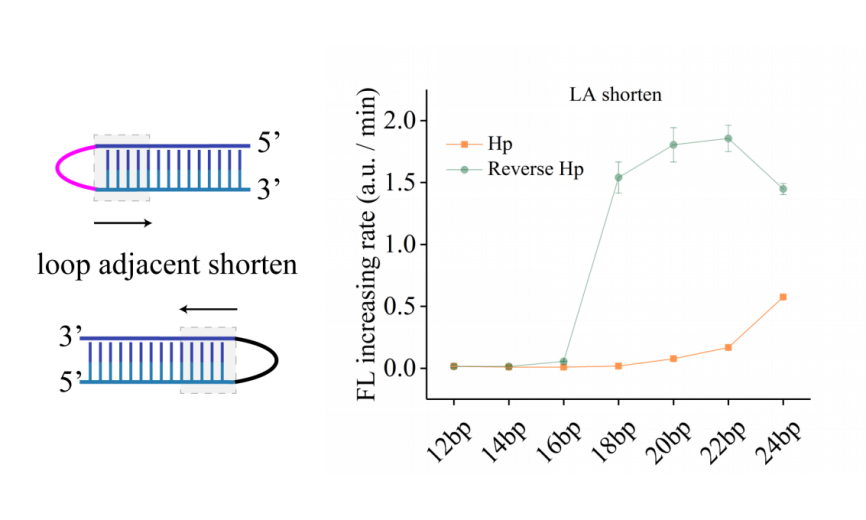


**Fig. S8.** Comparison of loop adjacent shorten of the hairpin-structured substrate on the activation of Cas12a between hairpin and reverse hairpin. Represented as fluorescence increasing rate, Error bar, SD, n=3.


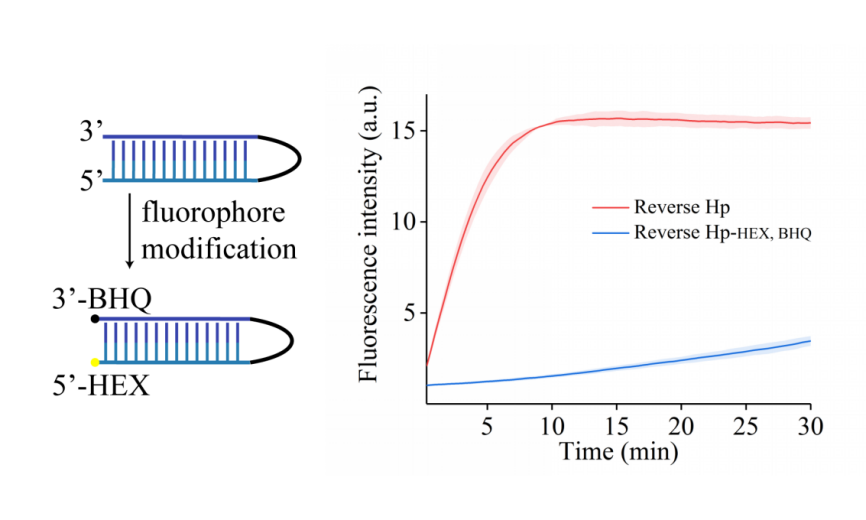


**Fig. S9.** The real-time fluorescence curves of the cleaved reporter for trans-cleavage cleavage activated by reverse hairpin and fluorophore-modified reverse hairpin: terminal modification of HEX and BHQ at reverse hairpin significantly inhibits the activation of Cas12a. Error bar, SD, n=3.


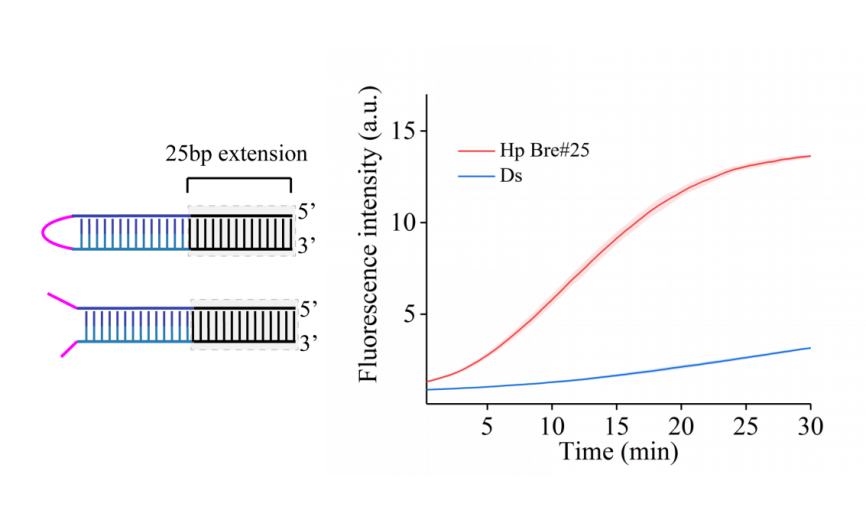


**Fig. S10.** The real-time fluorescence curves of the cleaved reporter for trans-cleavage cleavage activated by 25bp-extended dsDNA (Ds) and hairpin (Hp Bre#25) at end of the hairpin. Error bar, SD, n=3.


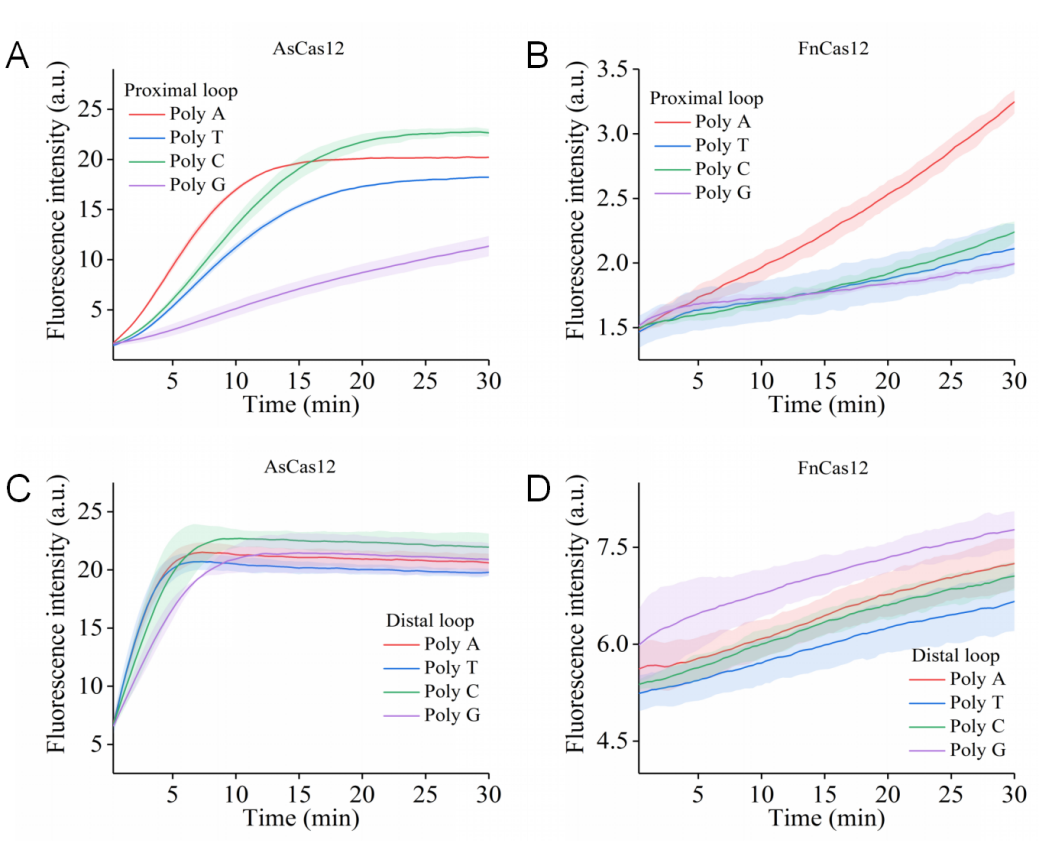


**Fig. S11.** The real-time fluorescence curves of the cleaved reporter for trans-cleavage cleavage of different Cas12a orthologs (AsCas12a, FnCas12a) activated by proximal loop hairpin (A-B) and distal loop hairpin (C-D) with 8-nt poly A/T/C/G loop. The activation result of LbCas12a is shown in Fig. 3B. Error bar, SD, n=3.


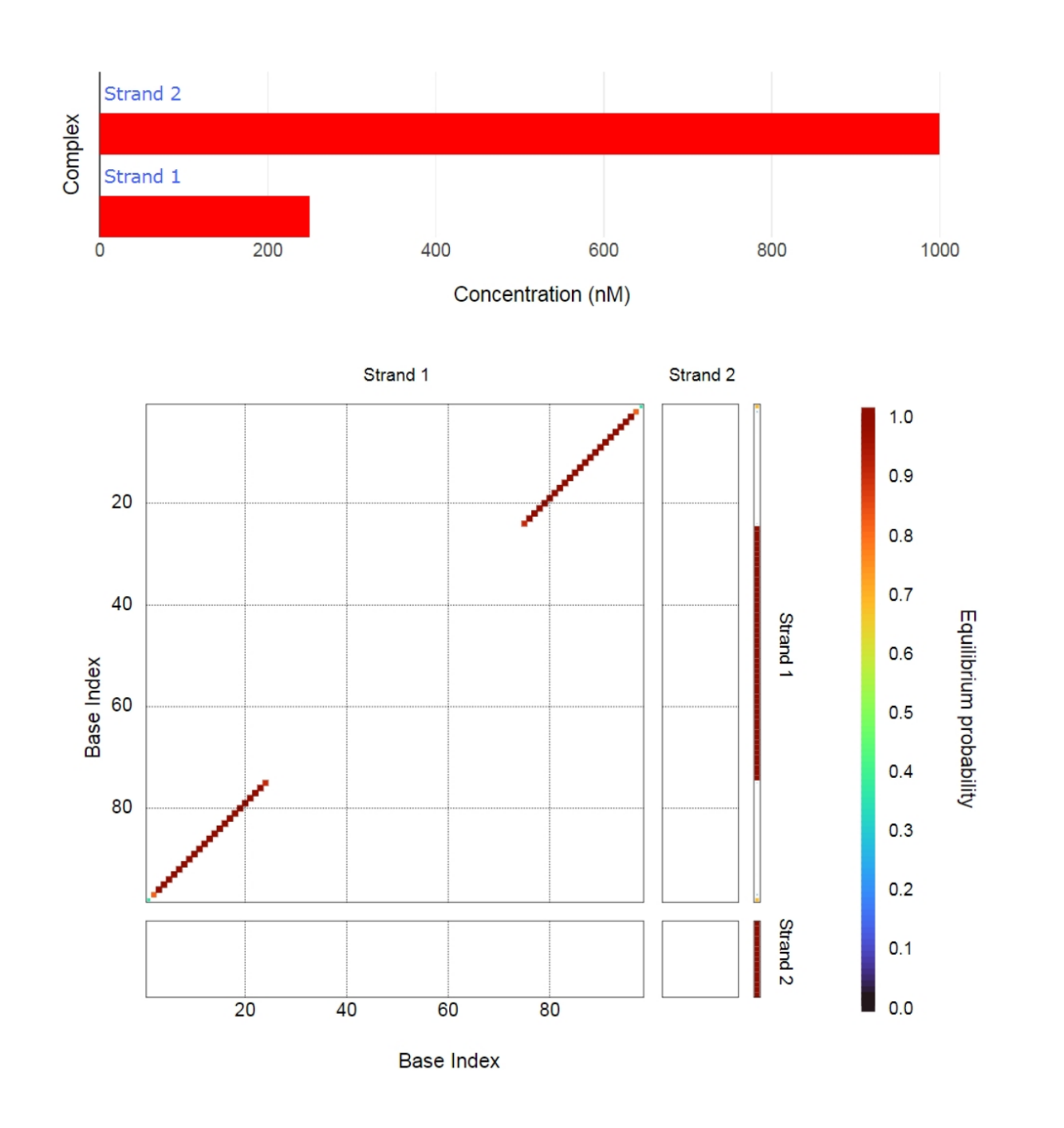


**Fig. S12.** The binding of a hairpin with a 50-nt poly-A loop and 15-nt poly-T reporter was analyzed by NUPACK. Strand 1 represents the hairpin substrate, and the substrates used in the experiment were all 250 nM. Strand 2 represents the reporter, and the reporter used in the experiment was 1 μM. The results in NUPACK show that even after mixing, the two still exist separately and there is no composite of strand 1 and strand 2. There was also no crossover in the equilibrium probability.

Theoretically, once binding occurs between reporters and hairpin with poly-A loop, the duplex resistance to trans-cleavage activity would prevent the reporters from being cleaved. On the contrary, proximal and distal poly-A loop hairpins both showed trans-cleavage activity, indicating that no binding occurred at this stage. Further analysis in NUPACK proved the conjecture, that even a hairpin with 50-nt poly-A loop did not bind to 15-nt poly(T) ssDNA.


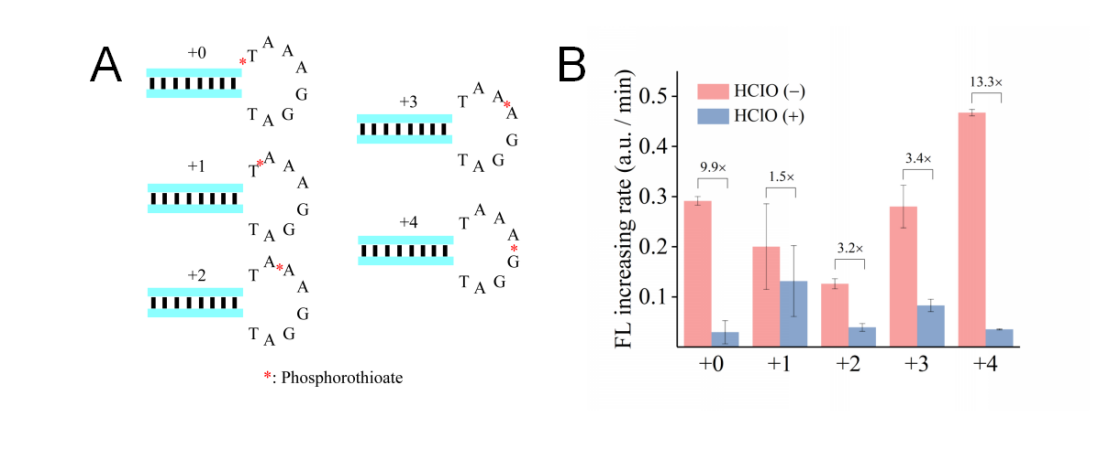


**Fig. S13.** (A) Schematic diagram of phosphorothioate modification of varying positions in the hairpin loop. (B) Optimization of phosphorothioate modification sites in the hairpin loop. Represented as fluorescence increasing rate. Error bar, SD, n=3.


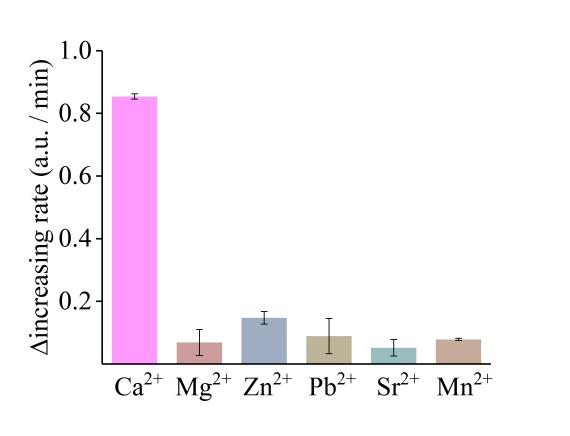


**Fig. S14.** Selective study for Ca^2+^, Mg^2+^, Zn^2+^, Pb^2+^, Sr^2+^ and Mn^2+^. The concentration of Ca^2+^ in the mixture was 100 nM, and the concentration of non-specific molecules was 1 μM. Represented as △fluorescence increasing rate, calculated by subtracting the fluorescence increasing rate of adding each molecule from the fluorescence increasing rate of the original hairpin substrate. Error bars, SD, n=3.


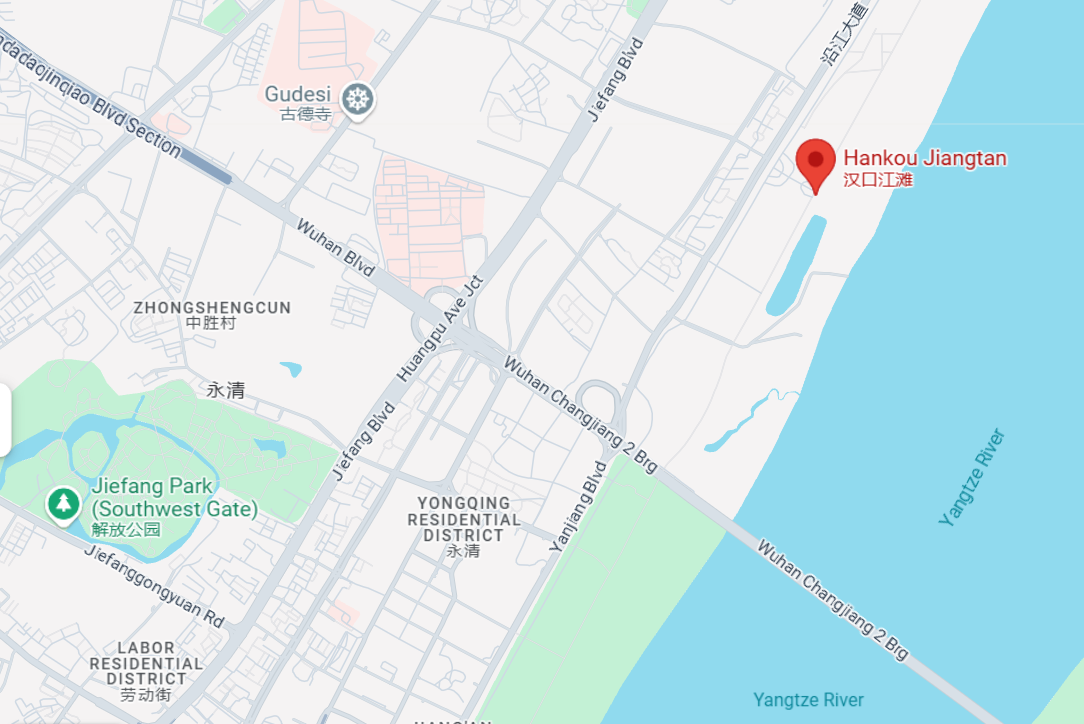


**Fig. S15.** The actual water sample collection location from the Yangtze River.


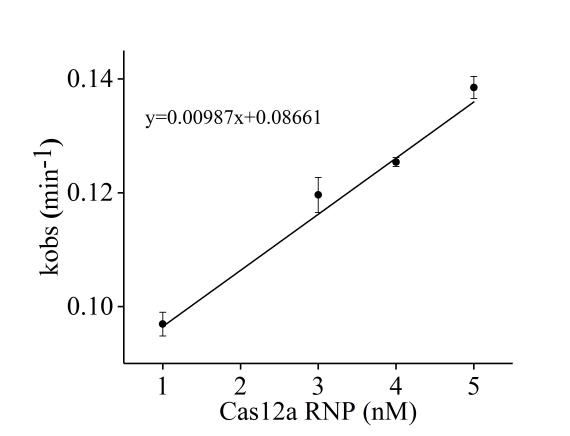


**Fig. S16.** The standard curve of k_obs_ vs [RNP] for dissociation constant. Error bar, SD, n=3.


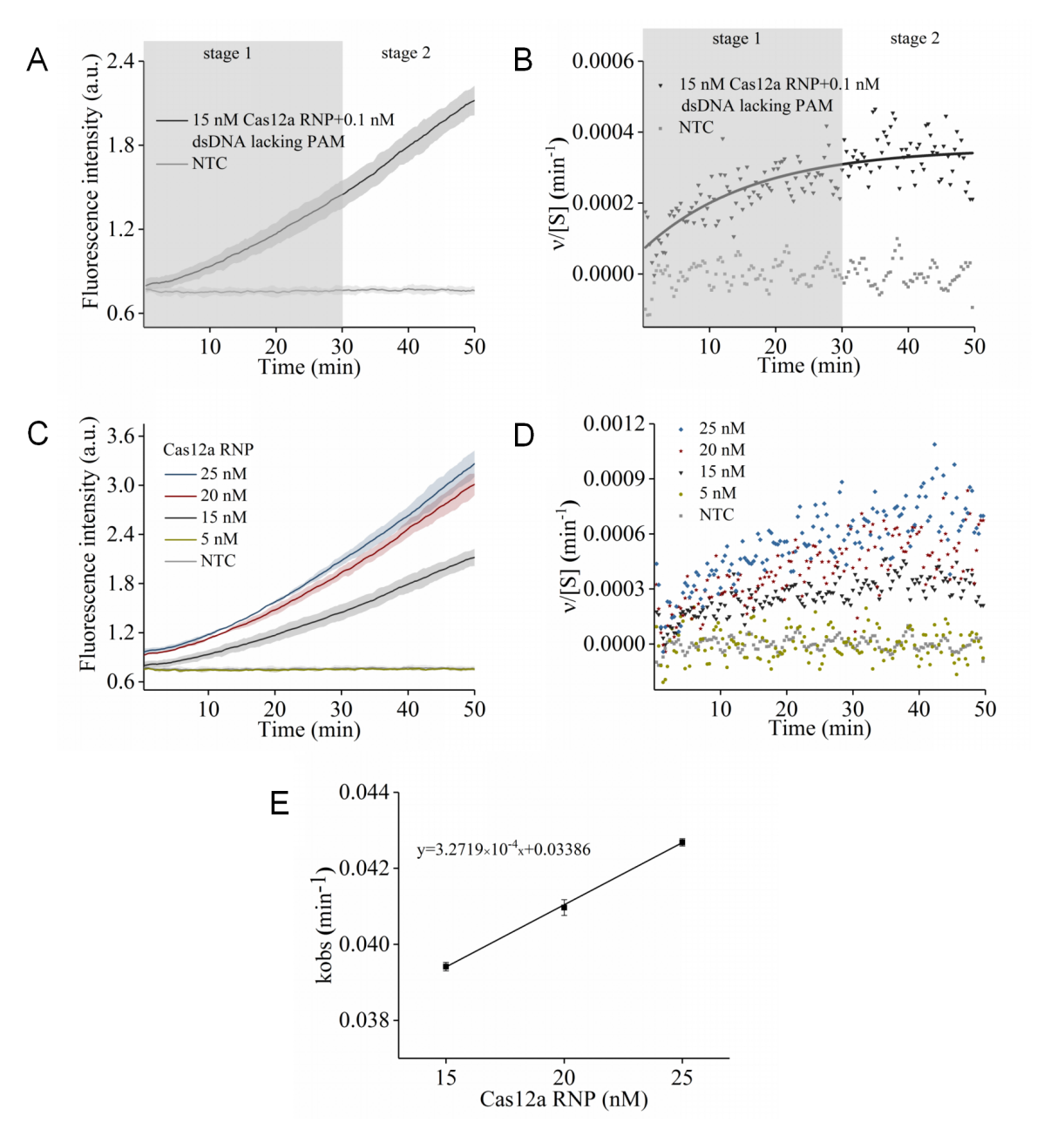


**Fig. S17.** (A) The real-time fluorescence curve of the cleaved reporter after the combination of RNP and activator of dsDNA lacking PAM: nonlinear phase of stage 1 and linear phase of stage 2. (B) The activator binding and LbCas12a activation kinetics. (C) The real-time fluorescence curve of the cleaved reporter after the combination of RNP and different concentrations of activator of dsDNA lacking PAM. (D) The activator binding and LbCas12a activation kinetics measured by different concentrations of RNP. (E) The standard curve of k_obs_ vs [RNP] for dissociation constant. Error bar, SD, n=3.


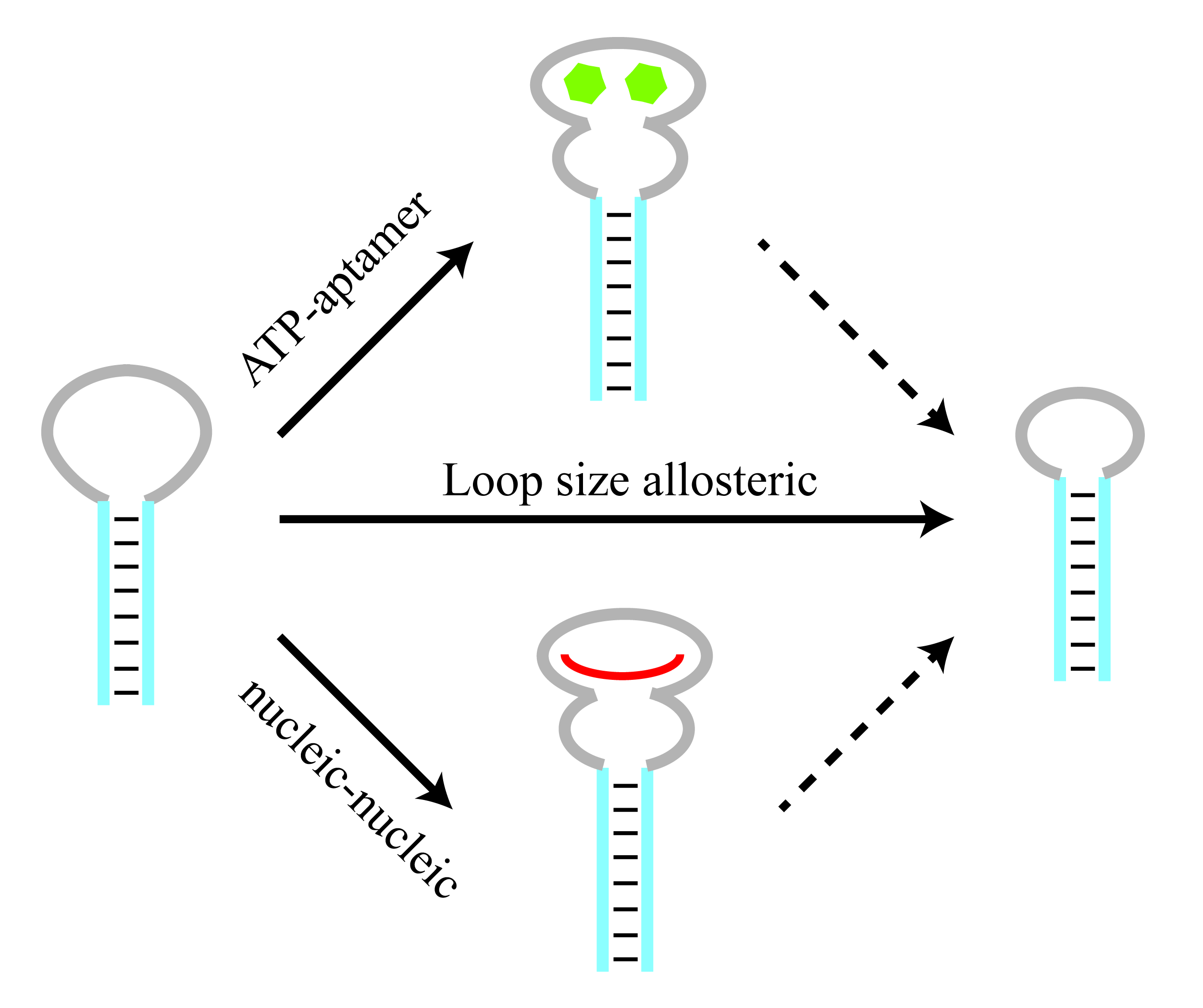


**Fig. S18.** Schematic diagram of steric allosteric changes of hairpin substrates by binding forces of ATP-aptamers and nucleic acid-nucleic acid.

References

1. Ma,J.Y., Liu,B., Raza,S., Jiang,H.X., Tang,A.N. and Kong,D.M. (2023) CRISPR/Cas12a-based hypochlorous acid and myeloperoxidase biosensors designed on RESET effect. Sensor. Actuat. B-Chem., 376, 133000.
2. Ge,X.G., Lou,Y.H., Su,L.C., Chen,B., Guo,Z.Y., Gao,S., Zhang,W.M., Chen,T., Song,J.B. and Yang,H.H. (2020) Single wavelength laser excitation ratiometric NIR-II fluorescent probe for molecule imaging in vivo. Anal. Chem., 92, 6111-6120.
3. Yang,L.L., Li,L.F., Liu,R.Y., Xie,C.G., Zhao,J., Chang,W.G., Chen,L.J., Yan,Y.H., Zhang,N.N., Zhang,W., Liu,B.H. and Yang,L. (2024) Cationic fluorescent carbon dots with solution ultra-stability and its rapid/on-site sensing application for HClO. Talanta, 267, 125137.
4. Ji,M.X., Wei,Y.C., Ye,Z., Hong,X.Q., Yu,X.X., Du,R., Li,Q., Sun,W. and Liu,D.B. (2024) In vivo fluorescent labeling of foam cell-derived extracellular vesicles as circulating biomarkers for in vitro detection of atherosclerosis. J. Am. Chem. Soc., 146, 10093-10102.
5. Wang,L., Hiblot,J., Popp,C., Xue,L. and Johnsson,K. (2020) Environmentally sensitive color-shifting fluorophores for bioimaging. Angew. Chem. Int. Ed., 59, 21880-21884.
6. Cheng,R.M., Li,Z.L., Chang,P.J., Shan,S.Y., Jiang,X.H., Hu,Z.X., Zhang,B., Zhao,Y. and Ou,S.J. (2025) Enhanced intracellular calcium detection using dopamine-modified graphene quantum dots with dual emission mechanisms. Spectrochim. Acta A., 328, 125475.
7. Li,C.B., Chen,P.F., Wang,Z.P. and Ma,X.Y. (2021) A DNAzyme-gold nanostar probe for SERS-fluorescence dual-mode detection and imaging of calcium ions in living cells. Sensor. Actuat. B-Chem., 347, 130596.
8. Peng,L.P., Yuan,G.Q., Ding,H.Y., Tan,L.B., Yang,Q.M. and Zhou,L.Y. (2021) Engineering a near-infrared nanosensor based on supramolecular self-assembly for Ca2+ detection and imaging in living cells and mice. Sensor. Actuat. B-Chem., 332, 129539.
